# Novel Insights into the Antimicrobial and Antibiofilm Activity of Pyrroloquinoline Quinone (PQQ); *In Vitro*, *In Silico*, and Shotgun Proteomic Studies

**DOI:** 10.3390/biom14081018

**Published:** 2024-08-16

**Authors:** Mai M. Labib, Alaa M. Alqahtani, Hebatallah H. Abo Nahas, Rana M. Aldossari, Bandar Fahad Almiman, Sarah Ayman Alnumaani, Mohammad El-Nablaway, Ebtesam Al-Olayan, Maha Alsunbul, Essa M. Saied

**Affiliations:** 1 Department of Bioinformatics, Agricultural Genetic Engineering Research Institute (AGERI), Agricultural Research Centre (ARC), Cairo 12619, Egypt; mailabib@ageri.sci.eg; 2 Department of Pharmaceutical Sciences, Faculty of Pharmacy, Umm Al-Qura University, Makkah 21955, Saudi Arabia; amqahtani@uqu.edu.sa; 3 Zoology Department, Faculty of Science, Port Said University, Port Said 42526, Egypt; heba.hassan@sci.psu.edu.eg; 4 Department of Pharmacology and Toxicology, College of Pharmacy, Prince Sattam Bin Abdulaziz University, Al-Kharj 11942, Saudi Arabia; r.alsaffar@psau.edu.sa; 5 Biology Department, College of Science, Al-Baha University, Al Bahah 65779, Saudi Arabia; balmiman@bu.edu.sa; 6 Department of Medical Microbiology, Faculty of Medicine, University of Jeddah, Jeddah 23218, Saudi Arabia; anomani@uj.edu.sa; 7 Department of Basic Medical Sciences, College of Medicine, AlMaarefa University, P.O. Box 71666, Riyadh 11597, Saudi Arabia; mnablawi@um.edu.sa; 8 Department of Medical Biochemistry, Faculty of Medicine, Mansoura University, Mansoura 35516, Egypt; 9 Department of Zoology, College of Science, King Saud University, Riyadh 11451, Saudi Arabia; eolayan@ksu.edu.sa; 10 Department of Pharmaceutical Sciences, College of Pharmacy, Princess Nourah Bint Abdulrahman University, P.O. Box 84428, Riyadh 11671, Saudi Arabia; maalsonbel@pnu.edu.sa; 11 Chemistry Department, Faculty of Science, Suez Canal University, Ismailia 41522, Egypt; 12 Institute for Chemistry, Humboldt Universität zu Berlin, 12489 Berlin, Germany

**Keywords:** pyrroloquinoline quinone, antimicrobial agents, antibacterial activity, antifungal activity, MRSA, antibiofilm activity, TEM analysis, shotgun proteomic analysis, molecular modeling

## Abstract

Microbial infections pose a significant global health threat, affecting millions of individuals and leading to substantial mortality rates. The increasing resistance of microorganisms to conventional treatments requires the development of novel antimicrobial agents. Pyrroloquinoline quinone (PQQ), a natural medicinal drug involved in various cellular processes, holds promise as a potential antimicrobial agent. In the present study, our aim was, for the first time, to explore the antimicrobial activity of PQQ against 29 pathogenic microbes, including 13 fungal strains, 8 Gram-positive bacteria, and 8 Gram-negative bacteria. Our findings revealed potent antifungal properties of PQQ, particularly against *Syncephalastrum racemosum*, *Talaromyces marneffei*, *Candida lipolytica*, and *Trichophyton rubrum*. The MIC values varied between fungal strains, and *T. marneffei* exhibited a lower MIC, indicating a greater susceptibility to PQQ. In addition, PQQ exhibited notable antibacterial activity against Gram-positive and -negative bacteria, with a prominent inhibition observed against *Staphylococcus epidermidis*, *Proteus vulgaris*, and MRSA strains. Remarkably, PQQ demonstrated considerable biofilm inhibition against the MRSA, *S. epidermidis*, and *P. vulgaris* strains. Transmission electron microscopy (TEM) studies revealed that PQQ caused structural damage and disrupted cell metabolism in bacterial cells, leading to aberrant morphology, compromised cell membrane integrity, and leakage of cytoplasmic contents. These findings were further affirmed by shotgun proteomic analysis, which revealed that PQQ targets several important cellular processes in bacteria, including membrane proteins, ATP metabolic processes, DNA repair processes, metal-binding proteins, and stress response. Finally, detailed molecular modeling investigations indicated that PQQ exhibits a substantial binding affinity score for key microbial targets, including the mannoprotein Mp1P, the transcriptional regulator TcaR, and the endonuclease PvuRTs1I. Taken together, our study underscores the effectiveness of PQQ as a broad-spectrum antimicrobial agent capable of combating pathogenic fungi and bacteria, while also inhibiting biofilm formation and targeting several critical biological processes, making it a promising therapeutic option for biofilm-related infections.

## 1. Introduction

Bacterial pathogens pose a very high risk to public health as a result of the frequent overuse of antibiotics that lead to the emergence of antibiotic-resistant microbial strains [1]. At least 700,000 deaths each year worldwide occur from drug-resistant diseases, and by 2050, this number is expected to rise to 10 million, making drug-resistant diseases the main cause of death, surpassing diabetes, heart disease, and cancer [2]. In 2019, infections caused by antibiotic-resistant bacteria resulted in an estimated 1.27 million deaths, which is more than deaths caused by HIV and malaria, 864,000 and 643,000, respectively [3]. The medical cost per patient with antibiotic-resistant illness is expected to be up to USD 29,069, and infections are frequently severe and lethal [4,5]. Bacterial infections become more difficult to cure when the disease-causing organism no longer responds to antibiotics [6]. Therefore, the effectiveness of therapeutic procedures requiring the use of antibiotics is seriously threatened due to the slow pace of antibiotic research and development [7,8]. The most common resistant strains are methicillin-resistant *Staphylococcus aureus* (MRSA), and multidrug-resistant *Pseudomonas aeruginosa*, *Streptococcus pneumoniae*, *Escherichia coli*, *Klebsiella pneumoniae*, and *Acinetobacter baumannii* [3]. Multidrug-resistant *P. aeruginosa* is known for its association with healthcare-associated infections, especially among immunosuppressed individuals. *S. pneumoniae* poses a challenge due to its resistance to multiple classes of antibiotics, which compromises the effective treatment of respiratory infections. *E. coli* and *K. pneumoniae*, both found in the gut microbiota, have become alarming as they exhibit resistance to a variety of antibiotics, including those commonly used for urinary tract and bloodstream infections. *A. baumannii*, prevalent in healthcare settings, demonstrated resistance to numerous antibiotics, leading to severe infections [9,10].

In recent times, the occurrence of opportunistic fungal diseases has led to a staggering estimate of 1.5 million deaths per year, mainly affecting individuals undergoing immunosuppressive treatments [11,12,13]. The COVID-19 crisis has brought about a host of challenges, including a notable increase in fungal infections among patients. Of particular concern is the emergence of antibiotic-resistant fungal infections, which have become an important issue during this period. Furthermore, the excessive use of antifungal agents in combating severe cases of COVID-19 has contributed to the development of drug-resistant fungal strains. Although these medications are included in therapeutic regimens, their misuse or overuse has led to the emergence of drug-resistant fungal species, thus limiting the efficacy of current treatment options [14,15]. The limited number of available antifungal classes for treating fungal infections, including polyenes, azoles, and echinocandins, poses a significant challenge when resistance to any one of these classes emerges. When a particular antifungal class becomes ineffective due to resistance, the available treatment options are significantly reduced [16]. The formation of drug-resistant fungal strains has been also reported in various fungal species including *Aspergillus terreus*, *Candida*, and *Fusarium* species that have developed resistance to polyenes, azoles, and echinocandins, respectively [17]. Numerous mechanisms of drug resistance have been discovered in fungi, including biofilm formation, target incompatibility, overexpression of drug transporters, and stimulation of stress response pathways [18,19].

Despite the fact that a variety of antibiotics with innovative molecules are currently being developed, more therapeutic alternatives and techniques are required for the treatment of infections. Since herbal remedies function similarly to antibiotics in that they either kill bacteria or hinder their replication, they can be used as therapeutic solutions for resistant bacterial strains [20,21]. Pyrroloquinoline quinone (4,5-dihydro-4,5-dioxo-1H-pyrrolo [2,3-f]quinolone-2,7,9-tricarboxilic acid, PQQ) is a noncovalently bound quinone-containing molecule that is found in plants, yeast, and some bacteria [22,23]. PQQ is found in vegetables, fruits, and beverages, including parsley, potatoes, carrots, cabbage, papaya, kiwi, banana, and green tea [24]. PQQ is a crucial cofactor involved in many cellular processes, including metabolism, division, and senescence. PQQ is used by bacterial and mammalian dehydrogenases and oxidases to enhance NADH oxidation to NAD+ and pyruvate production [25]. In addition, numerous possible health advantages of PQQ supplementation have been reported in studies, including increased metabolic flexibility and protection of the immune and nervous systems [22,26]. In addition to its role as a cofactor, PQQ supplementation has been the subject of numerous studies, which have reported various potential health benefits. One such benefit is the increase in metabolic flexibility, which refers to the ability of an organism to efficiently switch between different fuel sources for energy production. PQQ has been suggested to enhance this metabolic flexibility, potentially leading to improved energy metabolism and overall metabolic health. Furthermore, PQQ supplementation has been associated with protective effects on the immune system and nervous system [27]. Studies have suggested that PQQ may support immune function by reducing inflammation and oxidative stress, thus contributing to a healthier immune response. Additionally, PQQ has demonstrated neuroprotective properties, including the promotion of nerve growth factor synthesis and the prevention of neuronal damage. The multifaceted activity of PQQ as a cofactor and its potential health benefits make it a fascinating compound of interest in cellular biology and human health research [28]. A previous study further provided compelling evidence supporting the therapeutic benefits of PQQ in the context of pre-eclampsia by simultaneously modulating both the inflammatory and antioxidant pathways [29]. Overall, the diverse range of activities and potential health benefits of PQQ make it an intriguing subject of study in cellular biology and human health research.

For the first time, to the best of our knowledge, this study aimed to test the antimicrobial activity of PQQ against 29 pathogenic microbes: 13 fungal strains, 8 Gram-positive bacteria, and 8 Gram-negative bacteria, and we assessed the MIC/MBC for these microbes. Furthermore, we assessed the antibiofilm activity of PQQ in a panel of bacterial strains and explored its effect on the bacterial cells as well as the intracellular compartments by conducting transmission electron microscopic studies. Finally, we performed extensive *in silico* and shotgun proteomic studies to unveil the mode of action of the antimicrobial activity of PQQ.

## 2. Materials and Methods

### 2.1. Reagents and Chemicals

All chemicals and reagents used in this study were acquired from Sigma-Aldrich (Saint Louis, MO, USA), Across, or TCI (Chuo-ku, Tokyo, Japan) in a high grade (>95%).

### 2.2. Microbial Strains

A total of 29 pathogenic microbes were used in the study, divided into 13 fungal strains, 8 Gram-positive bacteria and 8 Gram-negative bacteria. Fungal strains included *Aspergillus fumigatus* (RCMB 002008), *A. niger* (RCMB 002005), *Candida albicans* RCMB 005003 (1) ATCC 10231, *Penicillium aurantiogriseum* IMI 89372, *Syncephalastrum racemosum* RCMB 016001 (1), *Talaromyces marneffei* (RCMB 001022), *Cryptococcus neoformas* RCMB 0049001, *Candida lipolytica* RCMB 005007 (1), *Penicillium expansum* RCMB 001001 (1) IMI 28169, *Penicillium italicum* RCMB 001018 (1) IMI 193019, *Fusarium moniliform* (RCMB 008005), *Trichophyton rubrum* (RCMB 025002), and *Geotrichum candidum* (RCMB 041001). The Gram-positive bacteria included *Staphylococcus aureus* ATCC 25923, *Bacillus subtilis* RCMB 015 (1) NRRL B-543, *Bacillus cereus* RCMB 027 (1), *Staphylococcus epidermidis* RCMB 009 (2), *Micrococcus* sp. RCMB 028 (1), *Streptococcus mutants* RCMB 017 (1) ATCC 25175, Methicillin-Resistant *Staphylococcus aureus* (MRSA) ATCC 4330, and *Enterococcus faecalis* (ATCC 29212). The Gram-negative bacterial strains used were *Enterobacter cloacae* RCMB 001 (1) ATCC 23355, *Salmonella typhimurium* RCMB 006 (1) ATCC 14028, *Escherichia coli* ATCC 25922, *Klebsiella pneumonia* RCMB 003 (1) ATCC 13883, *Proteus vulgaris* RCMB 004 (1) ATCC 13315, *Serratia marcenscens* 007001, *Pseudomonas aeruginosa* ATCC 27853, and *Porphyromonas gingivalis* RCMB 022001 (1) EMCC 1699.

### 2.3. Antimicrobial Activity Screening

Susceptibility tests were performed according to National Committee for Clinical Laboratory Standards (NCCLS) recommendations. The antimicrobial activity of PQQ was tested using the agar well diffusion technique in Mueller–Hinton agar plates (MHA) with wells of diameter 6 mm [30,31]. Each well was filled with 100 μL of PQQ sample (10 mg/mL), positive control (ketoconazole (100 μg/mL) for fungi and gentamycin (4 μg/mL) for bacteria), and negative control (DMSO). The inoculum suspension was prepared from colonies grown overnight on an agar plate and then inoculated into Mueller–Hinton broth and malt broth for bacteria and fungi, respectively. A sterile swab was immersed in the suspension and used to inoculate Mueller–Hinton agar plates. The test organisms were cultured aerobically for 48 h at 28 °C for fungi and for 24 h at 37 °C for bacteria in nutritional broth. The experimental assessments were conducted in triplicate and the results are expressed as mean ± SD. This ensured optimal growth conditions for the microorganisms. By conducting the antimicrobial activity screening in this manner, we adhered to standardized protocols and established a consistent approach to evaluate the effectiveness of PQQ against a variety of microorganisms.

### 2.4. MIC Determination

After the plates were inspected to observe the formation of an inhibition zone around the well, antimicrobial activity was determined by analyzing the MIC values. In this manner, PQQ was dissolved in dimethyl sulfoxide (DMSO) at various concentrations (10, 5, 2.5, 1.25, 0.625, and 0.313 mg/mL). The MIC value was then defined as the lowest concentration of PQQ at which no visible microbial growth was detected. This pivotal metric served as an indicator of the potent inhibitory effect of PQQ on the microorganisms tested. By determining the MIC values, we gained valuable insights into the effectiveness of PQQ as an antimicrobial agent.

### 2.5. MBC Determination

The minimal bactericidal concentration (MBC) serves as a predictor of bacterial eradication, reflecting the lowest concentration of PQQ required to eliminate 99.9% of the bacterial inoculum. After MIC testing, wherein the presence of PQQ demonstrated inhibition of visible growth, samples from MIC-negative wells were subjected to subculture in antimicrobial-free agar plates. Approximately 50 μL of solutions derived from test tubes without turbidity were aseptically transferred onto solid agar culture plates (HIME DIA, Maharashtra, India) using a swab and the spread plate method. Subsequently, the plates were incubated at 37 °C for 48 h to macroscopically assess the presence or absence of bacterial growth. The minimum concentration at which no bacterial proliferation was discerned, indicated by the absence of colonies, was reported as MBC [32].

### 2.6. MFC Assessment

The determination of *in vitro* fungicidal activities (minimum fungicidal concentrations or MFC) for PQQ followed previously established protocols [33]. After 72 h of incubation, 20 μL was extracted from wells exhibiting complete inhibition (100% or optical clarity), starting from the last positive well (growth similar to growth control well) and the growth control (drug-free medium) without agitating the well contents before extraction. This process involved a subculture of a single 20 μL volume on each plate of Sabouraud dextrose agar. The Sabouraud dextrose agar plates were then incubated at 35 °C until growth was observed in the growth control subculture, typically within 48 h. The MFC was determined as the lowest concentration of PQQ that resulted in either no growth or fewer than three colonies, achieving approximately 99 to 99.5% killing activity. It is important to note that the experiment was conducted in a dose-dependent manner. The concentrations used in the experiment were 1.25 mg/mL, 0.156 mg/mL, 1.25 mg/mL, 2.5 mg/mL, 0.156 mg/mL, 0.156 mg/mL, 0.652 mg/mL, and 0.625 mg/mL.

### 2.7. TEM (Transmission Electron Microscopy) Analysis

To prepare the bacterial cells for transmission electron microscopy (TEM), 24 h cultures grown on nutrient broth media were collected by centrifugation at 4000 rpm for 10 min. Subsequently, the collected cells were washed with distilled water. The samples were then fixed with 3% glutaraldehyde, rinsed in phosphate buffer, and postfixed in a potassium permanganate solution for 5 min at room temperature. Following the fixation steps, the samples underwent a dehydration process. This involved immersion of them in a series of ethanol solutions, starting at 10% and gradually increasing to 90%, with each alcohol dilution lasting 15 min. Finally, the samples were dehydrated in absolute ethanol for 30 min. After dehydration, the samples were infiltrated with epoxy resin and acetone in graded series until they were fully immersed in pure resin. Ultrathin sections were obtained on copper grids, which were then subjected to double staining with uranyl acetate and lead citrate. The stained sections were subsequently observed using a JEOL—JEM 1010 transmission electron microscope, Tokyo, Japan at 70 kV at the Regional Center for Mycology and Biotechnology (RCMB), Al-Azhar University [34,35,36,37].

### 2.8. Antibiofilm Inhibition Activity

In this study, eight bacterial species were used, including *S. epidermidis*, *MRSA*, *Micrococcus* sp., *E. cloacae*, *P. vulgaris*, *K. pneumoniae*, *S. typhimurium*, and *S. aureus*. These bacteria were cultured in a Mueller–Hinton broth (MHB) medium. To prepare the bacterial suspensions, they were diluted to a concentration of 5 × 10^5^ CFU / mL in fresh MHB medium supplemented with 0.2% glucose. For the experiment, 90 µL of each bacterial suspension was mixed with 10 µL of the PQQ compound at series concentrations (0.156, 0.652, 1.25, 2.5 mg/mL) in a 96-well plate. Subsequently, the plate was incubated for 24 h. After incubation, the medium was gently aspirated, and the biofilm was fixed with 100% methanol. The methanol was then removed, and the biofilm was allowed to air dry. Crystal violet staining was then performed in the wells, and the staining process lasted 30 min. The crystal violet stain was then removed, and the wells were rinsed three times with distilled water. After air drying, 95% ethanol was added to dissolve the biofilm and the absorbance at 595 nm was measured to quantify biofilm formation. To observe the inhibitory effect of PQQ on biofilm formation, SYTO9 staining was used. After incubation of the bacterial species with PQQ for 24 h, the biofilms were stained with SYTO9 in the dark for 30 min. This allowed us to visualize the inhibition of the biofilm caused by PQQ.

### 2.9. Shotgun Proteomics Analysis

#### 2.9.1. Treatments and Lysate Sample Preparation

To investigate the effect of PQQ on the dynamic range of proteins in *Staphylococcus epidermidis* and *Proteus vulgaris*, we conducted a proteomic analysis for the cellular proteins from both PQQ-treated and control samples of *Staphylococcus epidermidis* and *Proteus vulgaris* strains. In this regard, *S. epidermidis* and *P. vulgaris* were introduced into the designated liquid broth medium and allowed to reach the exponential growth phase by incubation at 37 °C and 200 rpm for 18 h (6 plates for negative and treated groups/strain, triplicate). After incubation, the treated group was subjected to PQQ (156.25 µg/mL) and incubated at the same conditions for an additional 18 h, while the control group was left without any treatment for the entire incubation process. Finally, the samples from the control and treated groups were recovered from the incubation and kept at −80 °C until the proceeding of proteomic analysis. The cellular proteins from samples were extracted by homogenization and centrifugation for 30 min at 10,000 rpm. The protein lysates, after tryptic digestion, were transferred to 30 kDa filters. Extraction was achieved by applying approximately 100 μL of a lysis solution (8 M urea, pH 8.5, 500 mM Tris HCl) onto the filters, followed by subsequent complete ultra proteases (Roche, Mannheim, Germany). Following incubation at 37 °C for 1 h with periodic vortexing, the samples were centrifuged at 12,000 rpm for 20 min. Lysates from all samples were evaluated using the BCA method (Pierce, Rockford, IL, USA) at 562 nm before the digestion process.

#### 2.9.2. Protein Tryptic Digest

The samples were processed in solution, where the protein pellets were treated with 200 mM 1,4-dithiothreitol (DTT) for 30 min to induce reduction. Subsequently, 10 mM iodoacetamide was applied to the alkylate cysteine residues and the resulting mixture was finally diluted with 100 mM Tris-HCl (pH 8.5) to a final concentration of 2 M. For endopeptidase digestion, modified porcine trypsin (MS grade, Sigma, Neustadt an der Weinstraße, Rhineland-Palatinate, Germany) was added to the samples and the resulting mixture was incubated at 37 °C in a thermo-shaker (600 rpm) for 16 h. The resulting peptide mixture was purified by using a stage tip, as previously detailed [38]. The peptides were quantified using the BCA method (Pierce, Rockford, IL, USA) at 562 nm prior to injection, with a concentration set at 1 µg/10 µL.

#### 2.9.3. Nano-LC MS/MS Examination

Analysis was carried out using an Orbitrap Fusion Lumos Tribrid mass spectrometer (Thermo Fisher Scientific, Waltham, MA, USA) as previously reported [38,39,40]. The peptide precursor scans, with 120,000 resolutions, were executed across the mass-to-charge ratio (*m*/*z*) spectrum, spanning 400 to 1800. The maximum ion injection time (IT) and automated gain control (AGC) were configured in auto-mode according to standard procedures. For tandem MS analysis, only peptides with charge states between 2 and 7 were considered. Isotopes were excluded and dynamic exclusion, with a mass tolerance of 10 ppm, was set to 30 s. MS2 scans were performed in the quadrupole, utilizing a 1.5 *m*/*z* isolation window. The experimental protocol included applying higher-energy collisional dissociation (HCD) activation at a vibrant 30% collision energy. This was achieved through the rhythmic coordination of dynamic injection time mode and the reliable automatic gain control (AGC) target. The generated fragments were identified and registered using the rapid scanning rate within the linear ion trap at various scanning speeds. In profile mode, MS1 spectra were detected, whereas MS2 spectra were captured in centroid mode. Each sample was independently tested three times.

#### 2.9.4. Processing of Proteomics Data Analysis

Proteome Discoverer 1.4.3 (version 2.4.0.305, Thermo Scientific) was used to process proteomic data from raw files for the identification of peptides and proteins [41]. The databases used were the UniProt FASTA database (Canonical and Swissprot) of *Staphylococcus epidermidis* and *Proteus vulgaris*, containing 8236 and 11,240 entries, respectively, using the Sequest HT search engine and the label-free quantification method. All fully and semitryptic peptides were scrutinized with allowances for up to 2 missed cleavages, ensuring a peptide length ranging from a minimum of 6 to a maximum of 144 amino acids. The identification process involved setting an initial mass tolerance of 20 ppm for the precursor mass and 0.5 Da for the fragment mass. Cysteine carbamidomethylation (+57.02146 amu) was statically modified, while variable modifications included methionine oxidation (+15.995), pyrrolidone formation from carbamidomethylated C (−17.03 amu) and acetylation of protein N-terminal and K (+42.01 amu). Employing the Precolator, we utilized a target/decoy concatenated approach with validation based on the q-value to ensure robust results. To maintain stringency, a false discovery rate (FDR) of 1% was applied to peptides (minimum length of 7 amino acids) and proteins.

#### 2.9.5. Statistical Analysis

The quantification ratios used in this study were alveolar/control, embryonal/control, and alveolar/embryonal. Protein abundances were calculated using the summed abundance parameter. Quantification utilized unique razor peptides, with precursor abundance determined by intensity measurement. Normalization of the total amount of peptide was performed. The protein ratio calculation used a pairwise ratio based on a t-test (background-based) as a hypothesis test.

### 2.10. In Silico Molecular Docking Studies

The structure of the PQQ was obtained from the PubChem database (CID: 1024) to further assess its interactions with targeted microbes at the molecular level by conducting molecular docking simulations. Subsequently, the format was converted from sdf to pdbqt by Open Babel software (Version 2.3.1). For each microbe inhibited by PQQ, a target receptor was selected to study its interaction with PQQ. The receptors were obtained from the Protein DataBank (PDB) (ID: 3l1n, 4oq2 and 3kp4 for *T. marneffei*, *Proteus vulgaris* and *Staphylococcus epidermidis*, respectively). Each target receptor was prepared by removing water and other cocrystallized ligands and adding polar hydrogen atoms. Additionally, the grid box was adjusted to target the entire molecule with a grid spacing of 1Å between the set points [42]. The grid box was centered at 7.360, 15.139, and 44.372 at points 24, 10, and 28 on x, y, and z dimensions for *T. marneffei*, 16, 22, and 14 points at centers 40.975, 12.985, and 82.208 on the x, y, and z planes, respectively, for *Proteus vulgaris*, 24, 32, and 22 centered at −27.743, −39.581, and −1.746 on the x, y, and z axes, respectively, for *Staphylococcus epidermidis*. Docking simulations and binding affinity estimation were performed with AutoDock Vina [43]. To better understand the PQQ–protein interaction, the BIOVIA discovery studio was used to visualize the molecular interactions and detect the chemical bonds formed during the binding [44,45,46,47,48,49,50,51,52].

## 3. Results

### 3.1. Assessment of Antimicrobial Potential

#### 3.1.1. Antifungal Activity

**Figure 1:**
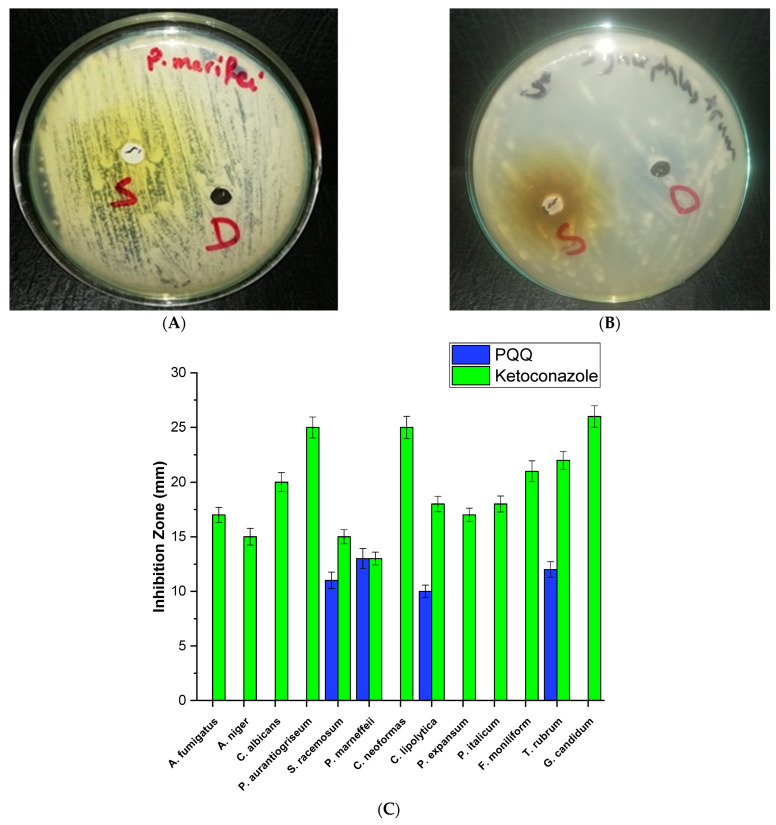
Growth inhibition zones of *T. marneffei* (**A**) and *T. rubrum* (**B**) after treatment with DMSO (D) and PQQ (S). (**C**) Antifungal activity of PQQ against various fungal strains.

The antifungal activity of PQQ was evaluated against a total of 13 different fungal strains belonging to various genera. The inhibition zone represents the area where the growth of the fungal strain is effectively suppressed, indicating the antifungal potential of the compound. Among the strains tested, PQQ exhibited considerable antifungal activity towards *S. racemosum*, *T. marneffei*, *C. lipolytica*, and *T. rubrum*. The highest antifungal activity was observed against *T. marneffei* with an inhibition zone of 13 mm, compared to the ketoconazole control used in the study (Figure 1). However, the antifungal activity of PQQ varied among the different strains tested. For example, compared to antifungal Ketoconazole, the inhibition zones resulting from PQQ treatment were smaller for strains such as *Syncephalastrum racemosum*, *Candida lipolytica*, and *Trichophyton rubrum* (Figure 1). This suggests that PQQ may be less effective against these particular fungi compared to ketoconazole. It should be mentioned that PQQ did not exhibit inhibitory effects on other species belonging to the genera *Aspergillus*, *Candida*, *Cryptococcus*, *Fusarium*, *and Geotrichum*.

#### 3.1.2. Antibacterial Activity against Gram-Positive Bacteria

**Figure 2:**
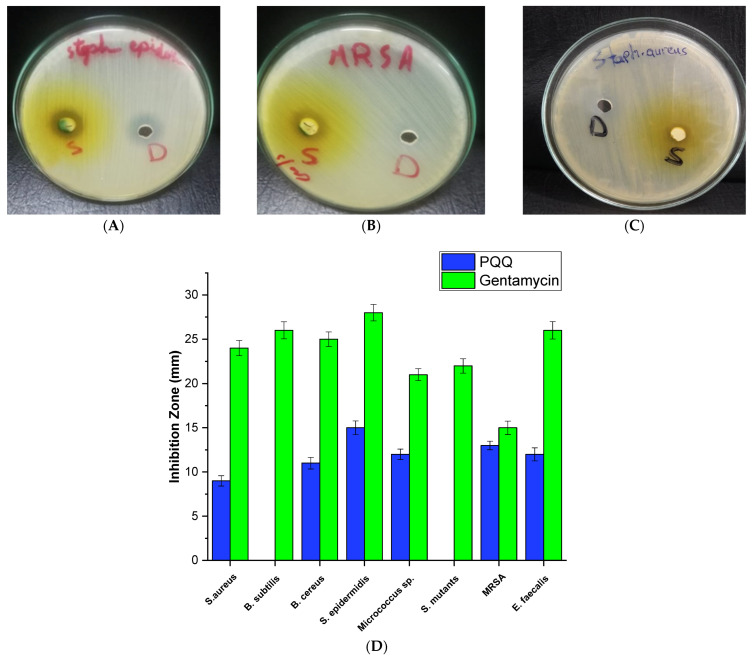
Growth inhibition zones of *S. epidermidis* (**A**), *MRSA* (**B**), and *S. aureus* (**C**) after treatment with DMSO (D) and PQQ (S). (**D**) Antibacterial activity of PQQ against various Gram-positive bacterial strains.

The antibacterial activity of PQQ was tested against eight different strains of Gram-positive bacteria. The results revealed varying degrees of inhibition against different strains. The most significant inhibition zone was observed for *Staphylococcus epidermidis RCMB 009* with 15 mm inhibition in diameter, a bacterium commonly found on the skin and mucous membranes. This finding suggests that PQQ could potentially be effective in combating infections caused by this particular strain (Figure 2). Additionally, PQQ exhibited a notable inhibitory effect against MRSA, a drug-resistant strain, with an inhibition zone measuring 13 mm. This result is particularly encouraging, as MRSA infections pose significant challenges in clinical settings (Figure 2). Similarly, an inhibition zone of 12 mm was observed for both *Micrococcus* sp. and *Enterococcus faecalis*, indicating that PQQ may have a broad-spectrum effect against these bacteria. However, PQQ treatment showed no significant inhibition compared to gentamycin toward target strains. For *Bacillus cereus*, a bacterium associated with food poisoning, a smaller inhibition zone of 11 mm was observed, suggesting a moderate impact of PQQ against this strain. Similarly, a relatively smaller inhibition zone of 9 mm was recorded for *S. aureus*. No visual inhibition was found against the *Bacillus subtilis* or *Streptococcus mutants*. Overall, these results indicate that PQQ exhibits antibacterial activity against selected Gram-positive bacterial strains, with notable inhibition zones observed for *S. epidermidis*, MRSA, *Micrococcus* sp, and *E. faecalis*.

#### 3.1.3. Antibacterial Activity against Gram-Negative Bacteria

**Figure 3:**
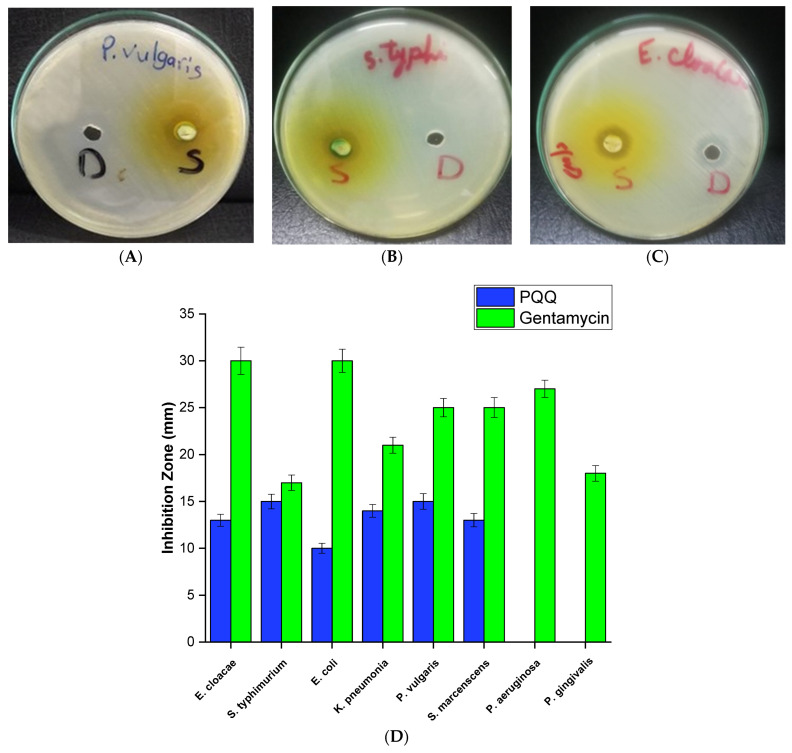
(**A**–**C**) Growth inhibition zones of *Proteus vulgaris* (**A**), *Salmonella typhimurium* (**B**), and *Enterobacter cloacae* (**C**) after treatment with DMSO (D) and PQQ (S). (**D**) Antibacterial activity of PQQ against various Gram-negative bacterial strains.

The inhibitory effects of PQQ were investigated in eight Gram-negative bacterial strains. Significant variations in the inhibition zones were observed. Among the strains examined, *Proteus vulgaris* RCMB 004 (1) ATCC 13315 and *Salmonella typhimurium* RCMB 006 (1) ATCC 14028 showed the largest inhibition zones of 15 mm, suggesting that PQQ has a strong inhibitory effect on the growth of these bacteria. Furthermore, PQQ exhibited considerable antibacterial activity against *Enterobacter cloacae* RCMB 001 (1) ATCC 23355, *Serratia marcenscens* 007001, and *K. pneumoniae* with inhibition zones of 13 and 14 mm, respectively (Figure 3), indicating that PQQ displays a wide-spectrum antibacterial activity against Gram-negative strains. However, the activity of PQQ did not surpass the inhibitory effect of gentamycin, as shown in Figure 3. Treatment with PQQ also revealed moderate inhibition zones of 10 mm against the *Escherichia coli* strain. On the other hand, PQQ demonstrated a nonsignificant activity against strains of *Pseudomonas aeruginosa* and *Porphyromonas gingivalis* strains. Together, these results indicate a considerable antibacterial effect of PQQ against Gram-negative strains.

### 3.2. Determination of MIC against Microbial Strains

#### 3.2.1. Evaluation of Antifungal Activity

**Figure 4:**
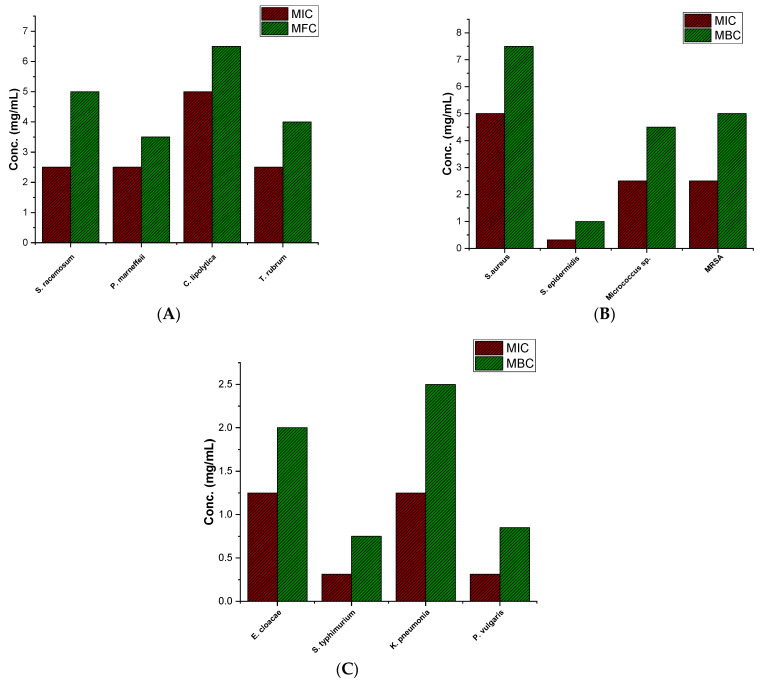
Minimum inhibitory concentration (MIC) and minimum microbial concentration of PQQ against a set of (**A**) fungal, (**B**) Gram-positive bacterial, and (**C**) Gram-negative bacterial strains.

During the investigation of PQQ antifungal activity, the minimum inhibitory concentration (MIC) was determined for selected fungal strains. Among the strains tested, PQQ exhibited an MIC value of 5 mg/mL toward *Candida lipolytica* to effectively inhibit its growth. This indicates that *Candida lipolytica* is relatively more resistant to PQQ’s antifungal effects, as a higher concentration was necessary to prevent its growth. In the other strains, including *S. racemosum*, *T. marneffei*, and *T. rubrum*, PQQ showed a lower MIC of 2.5 mg/mL, suggesting that comparatively lower concentrations of PQQ were sufficient to inhibit their growth. These results indicate that PQQ exhibits variable degrees of antifungal activity toward the investigated strains. Furthermore, we explored the minimum fungal concentration (MFC) of PQQ against this panel of fungal strains (*S. racemosum*, *T. marneffei*, *C. lipolytica*, and *T. rubrum*). The MFC values were determined by subjecting each strain to varying concentrations of PQQ and identifying the lowest concentration at which fungal growth was completely inhibited. The results of our investigation demonstrated the effectiveness of PQQ as an antifungal agent against the fungal strains tested. Our findings revealed that a minimum concentration of PQQ (5 mg/mL) was required to completely inhibit the growth of *S. racemosum*. Similarly, PQQ exhibited a lower MFC value of 3.5 mg/mL toward *T. marneffei*, indicating a higher susceptibility to PQQ. On the contrary, our investigations indicated a slightly higher MFC value (6.5 mg/mL) of PQQ toward *C. lipolytica*, suggesting a comparatively lower sensitivity to the inhibitory effects of PQQ. Lastly, PQQ demonstrated moderate susceptibility to *T. rubrum*, with an MFC value of 4 mg/mL (Figure 4). The observed variations in the MFC values among the different fungal strains can be attributed to inherent differences in their susceptibility to PQQ or variations in the mechanisms of action used by PQQ against these strains.

#### 3.2.2. Evaluation of Antibacterial Activity against Gram-Positive Bacteria

As shown in Figure 4, the results showed that PQQ exhibited limited antibacterial activity against four bacteria tested, as indicated by the high MIC values of 5 mg/mL for *S. aureus* and 2.5 mg/mL for *Micrococcus* sp. and MRSA. However, it should be noted that PQQ displayed a remarkably strong antibacterial effect against the Gram-positive bacterium *S. epidermidis*, inhibiting bacterial growth at a concentration of 312.5 μg/mL. This highlights the varying susceptibility of different bacteria to PQQ’s antibacterial properties. Although PQQ exhibited moderate antibacterial activity against *S. aureus*, *Micrococcus* sp., and MRSA, it demonstrated remarkable effectiveness in inhibiting the growth of *S. epidermidis* at a relatively lower concentration. Furthermore, we assessed the minimum bactericidal concentration (MBC) of PQQ against this set of Gram-positive bacterial strains (*S. aureus*, *S. epidermidis*, *Micrococcus* sp., and *MRSA*). The MBC values were determined by subjecting each strain to varying concentrations of PQQ and identifying the lowest concentration at which bacterial growth was completely inhibited. The results of our study revealed the potent antibacterial activity of PQQ against the tested Gram-positive bacterial strains. Among the strains examined, PQQ exhibited the lowest MBC value (1 mg/mL) toward *S. epidermidis*, indicating higher susceptibility to PQQ compared to the other tested Gram-positive bacteria. On the other hand, PQQ showed a relatively higher MBC value (7.5 mg/mL) toward *S. aureus*, indicating that a higher concentration of PQQ was required to completely inhibit the bacterial growth. Toward *Micrococcus* sp. and *MRSA*, PQQ showed MBC values of 4.5 mg/mL and 5 mg/mL, respectively, suggesting its moderate sensitivity (Figure 4). These findings highlight the potential of PQQ as an antibacterial agent against Gram-positive bacterial strains. The observed variations in MBC values among the different strains may be attributed to inherent differences in their susceptibility to PQQ or variations in the mechanisms of action through which PQQ exerts its antibacterial effects. Taken together, our results indicate that PQQ displays a considerable antimicrobial potential, as compared to reference drugs (Tables S1 and S2), suggesting further exploration in the mode of action toward microbial strains.

#### 3.2.3. Assessment of Antibacterial Activity against Gram-Negative Bacteria

As depicted in Figure 4, growth inhibition (MIC) of Gram-negative strains was observed at lower concentrations of PQQ, compared to that of Gram-positive strains. PQQ exhibited MIC of 1250 μg/mL toward *Enterobacter cloacae* and *K. pneumoniae*, indicating that PQQ was effective in hindering the growth of these strains even at relatively moderate concentrations. Interestingly, PQQ displayed significant inhibition of *Proteus vulgaris* and *S. typhimurium* with an MIC value of 312.5 μg/mL, suggesting that these strains are particularly susceptible to the antimicrobial effect of PQQ. These findings suggest that PQQ has potent antimicrobial properties toward Gram-negative bacteria, as lower concentrations of PQQ were effective in hindering the growth of these strains. We conducted further investigations to determine the minimum bactericidal concentration of PQQ required to completely inhibit the growth of a set of Gram-negative bacterial strains (*E. cloacae*, *S. typhimurium*, *K. pneumoniae*, *and P. vulgaris*). In this regard, the bacterial strains were subjected to different concentrations of PQQ, and the MBC values of PQQ toward the examined bacteria were assessed, which revealed the antibacterial activity of PQQ against the tested Gram-negative bacteria. In particular, PQQ exhibited a considerably low MBC value (0.75 mg/mL) toward *S. typhimurium*, indicating a higher susceptibility to PQQ compared to the other Gram-negative bacteria tested. In addition, PQQ showed a low MBC value of 0.85 mg/mL toward *P. vulgaris*, suggesting a significant sensitivity to PQQ. On the other hand, our findings revealed slightly higher MBC values (2 mg/mL and 2.5 mg/mL, respectively) for PQQ toward *E. cloacae* and *K. pneumoniae*, indicating moderate susceptibility to PQQ. These findings highlight the potential of PQQ as an effective antibacterial agent against Gram-negative bacterial strains. The observed variations in MBC values between the different strains could be attributed to inherent differences in their susceptibility to PQQ or variations in the mechanisms of action employed by PQQ against Gram-negative bacteria.

### 3.3. Assessment of Antibiofilm Activity

**Figure 5:**
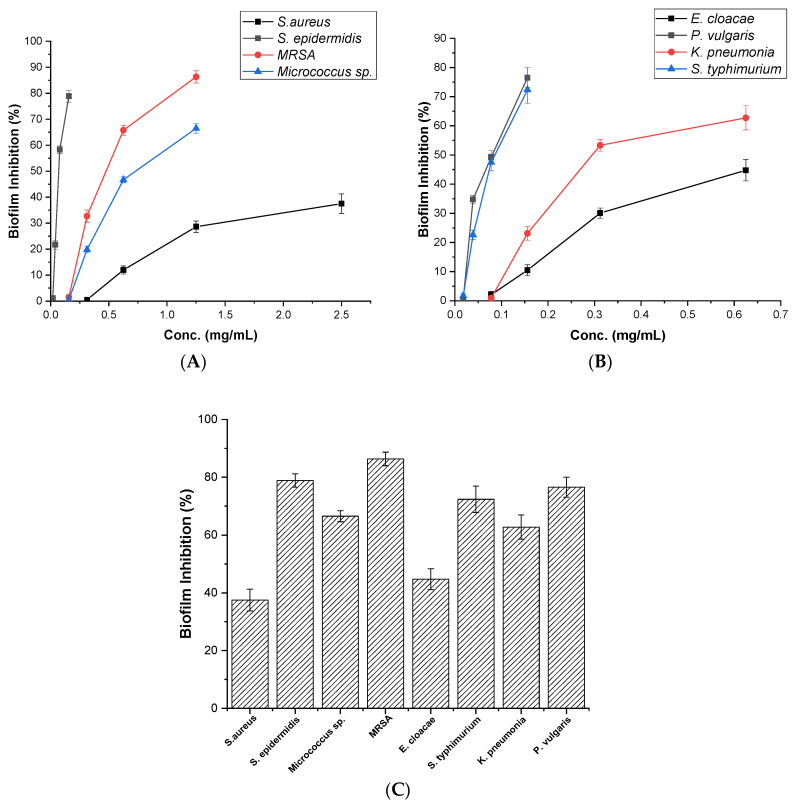
Antibiofilm inhibition activity of PQQ against a panel of Gram-negative and Gram-positive bacterial strains. (**A**) the dose-dependent biofilm inhibition (%) activity of PQQ against *S. aureus, S. epidermidis*, MRSA, and *Micrococcus* sp. (**B**) the dose-dependent biofilm inhibition (%) activity of PQQ against *E. cloacae*, *P. vulgaris*, *K. pneumoniae*, and *S. typhimunium* (**C**) the antibiofilm activity detected for PQQ against *S. aureus*, *S. epidermidis*, MRSA, *Micrococcus* sp., *E. cloacae*, *P. vulgaris*, *K. pneumoniae*, and *S. typhimunium*.

On the basis of these results, we further explored the mode of action of the antibacterial activity of PQQ by exploring its antibiofilm activity against various Gram-positive and Gram-negative bacteria, including *S. epidermidis*, MRSA, *Micrococcus* sp., *E. cloacae*, *P. vulgaris*, *K. pneumoniae*, and *S. typhimunium.* We employed a biofilm inhibition assay to assess PQQ’s inhibitory activity. Our results demonstrated significant inhibition activity of PQQ against the tested bacteria in a dose-dependent manner. Regarding its activity in Gram-positive bacteria, PQQ demonstrated substantial inhibitory activity against MRSA, achieving a remarkable inhibition rate of 86.31% at a concentration of 1.25 mg/mL. Additionally, PQQ exhibited high inhibitory activity against *S. epidermidis*, with an inhibition rate of 78.85% at a concentration of 0.156 mg/mL. Furthermore, PQQ showed considerable biofilm inhibition activity against *Micrococcus* sp., resulting in an inhibition rate of 66.48% at a concentration of 1.25 mg/mL. However, with *S. aureus*, PQQ demonstrated a limited inhibition rate of 37.55% at 2.5 mg/mL when exposed to PQQ as shown in (Figure 5). On the other hand, PQQ demonstrated significant inhibitory activity against Gram-negative bacteria, as shown in Figure 5. In particular, *P. vulgaris* exhibited a substantial reduction in biofilm formation, reaching 76.51% inhibition at a concentration of 0.156 mg/mL. Similarly, PQQ showed a high biofilm inhibitory activity against *S. typhimunium*, resulting in a noteworthy inhibition of 72.31% at a concentration of 0.156 mg/mL. In the case of *K. pneumoniae*, PQQ demonstrated considerable inhibition, with a rate of 62.7% at a concentration of 0.652 mg/mL. However, with *E. cloacae*, PQQ demonstrated a limited inhibition rate of 44.75% at 0.625 mg/mL when exposed to PQQ. Interestingly, the remarkable inhibitory effects of PQQ against MRSA, *S. epidermidis*, and *P. vulgaris* were evident even at a low concentration of 0.156 mg/mL. These findings indicate that PQQ has significant antimicrobial activity, specifically targeting the formation of biofilms by these bacterial species. These results suggest the potential utility of PQQ in combating biofilm-related infections caused by these organisms (Figure 5).

### 3.4. TEM Analysis

**Figure 6:**
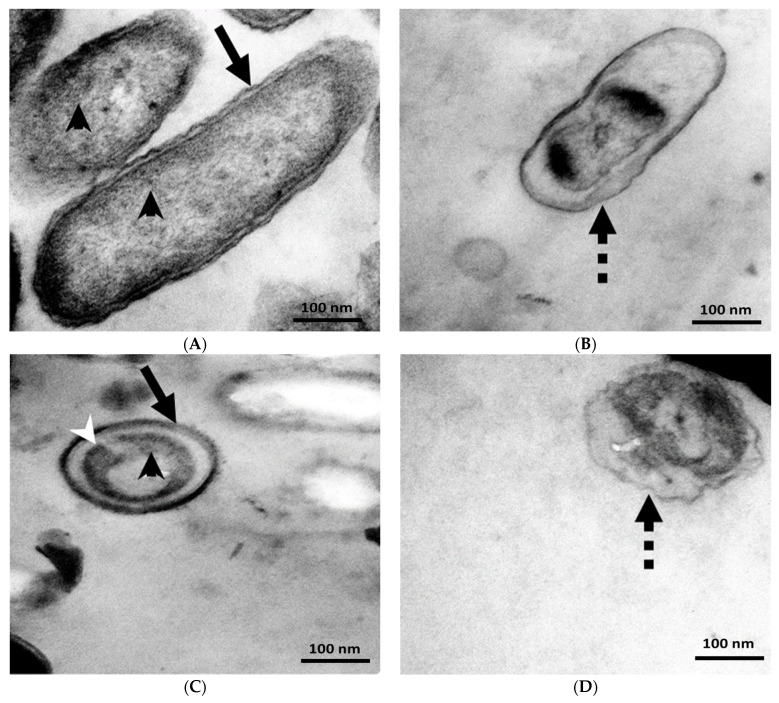
Effect of PQQ treatment on the morphology of *S. epidermidis* and *P. vulgaris*. (**A**) Untreated bacterium showing a normal straight rod-shaped bacterium. (**B**) PQQ-treated bacterium showing the damage of *P. vulgaris* membrane and bacterial compartments. (**C**) Untreated bacterium showing its normal spherical cocci morphology. (**D**) PQQ-treated bacterium showing damage to the membrane of *S. epidermidis* and bacterial compartments. Notice the intact cell membrane (arrows), disrupted cell membrane (dotted arrows), DNA (white arrowhead), and ribosomes (black arrowheads).

To investigate the PQQ-induced ultrastructural abnormalities in bacteria, additional investigations were conducted on microbes where PQQ exhibits high antibacterial activity. The aim was to explore the cellular destruction events and intracellular alterations in bacterial cells and shed light on the antibacterial effect and mode of action of PQQ at the nanoscale. TEM represents a powerful imaging technique that allows high-resolution visualization of cellular structures and ultrastructural changes. The experiments involved the preparation of bacterial samples for imaging, which typically included fixation, dehydration, embedding, sectioning, and staining. The experimental results revealed that PQQ treatment caused a series of events in *S. epidermidis* bacteria, leading to cell damage. These events included structural changes and disruptions in cell metabolism. Notably, the typical shape of the bacteria was visibly altered, indicating a loss of structural integrity. Furthermore, PQQ treatment induced intracellular stress within bacterial cells, contributing to the disruption of normal cellular processes and functions. This stress and associated damage resulted in aberrant morphology, cracking, and disruption of the cell membrane, compromising its integrity. Leakage of cytoplasmic content from the intracellular environment was also observed (Figure 6), indicating the damage caused by the treatment with PQQ. Consequently, the bacteria lost their typical shape. On the contrary, the untreated bacteria maintained their intact shape, highlighting that the shape change was specific to the cells treated with PQQ (Figure 6).

In the case of *P. vulgaris*, the application of PQQ led to significant effects on bacterial cells. The results indicated a complete loss of cell wall structure and function after PQQ treatment. This loss of cell wall integrity had several consequences. First, it resulted in a decrease in cell size, indicating that the bacterial cells became smaller as a result of the structural changes induced by PQQ. Furthermore, the cellular compartments within the bacteria experienced shrinkage, suggesting that the internal organization and morphology of the cells were disrupted. Moreover, PQQ treatment caused DNA agglomeration within bacterial cells. This implies that DNA, the genetic material of cells, was clumped together instead of being properly organized within the nucleus or nucleoid region (Figure 6). This disruption in DNA organization could have severe implications for the normal functioning and replication of bacterial cells. Furthermore, the presence of numerous ribosomes, which are responsible for protein synthesis, in the untreated bacterium disappeared with treatment with PQQ (Figure 6). This suggests that PQQ treatment had a detrimental effect on ribosomes, possibly inhibiting their function or causing their degradation. Loss of ribosomes would alter protein synthesis and could lead to a general impairment of cellular processes. As a consequence of these structural and functional disruptions, bacterial cells experienced local stress and cytosol leakage. Local stress refers to the internal stress experienced by cells as a result of the damage caused by PQQ. This stress likely affected various cellular processes and contributed to the overall deterioration of cells. Cytosol leakage refers to the release of the cell’s cytoplasmic contents into the surrounding environment, indicating severe damage to the cell membrane.

### 3.5. Shotgun Proteomic Analysis

**Figure 7:**
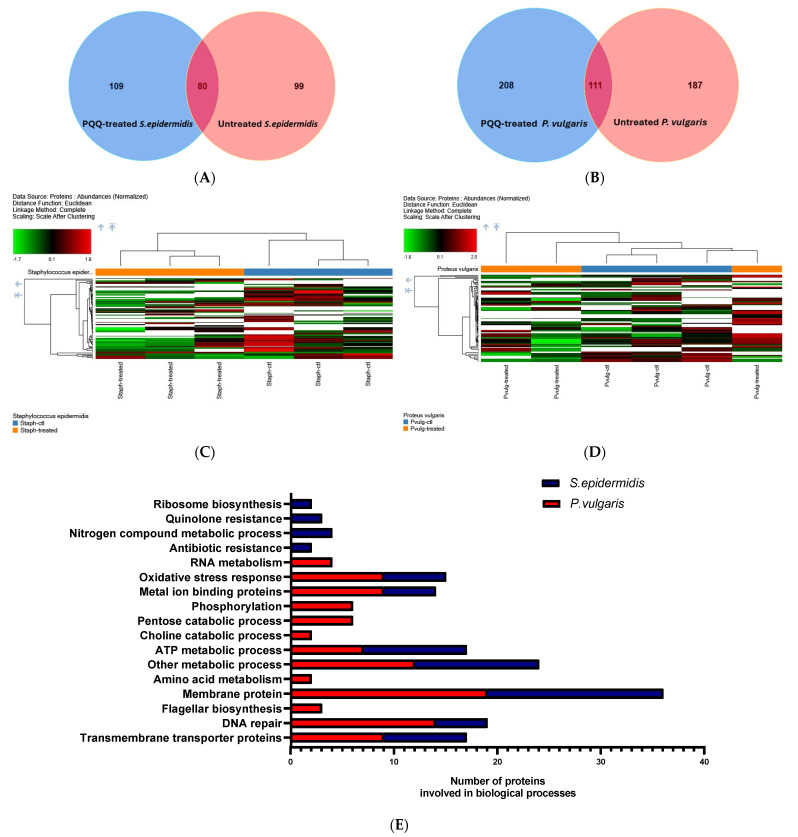
Evaluation of the proteomic profile of *P. vulgaris* and *S. epidermidis* bacteria after treatment with PQQ. (**A**,**B**) Venn diagram showing the proteins detected in PQQ treated and untreated *P. vulgaris* and *S. epidermidis* bacteria. (**C**,**D**) Heatmap of the protein abundance normalized after clustering in bacteria treated with PQQ and untreated with *P. vulgaris* and *S. epidermidis* (white blocks showed the absence of proteins). (**E**) Representative biological processes were identified from the functional cluster analysis of the unique proteins detected in PQQ-treated *P. vulgaris* and *S. epidermidis* bacteria.

To further gain more insights into the mode of action of PQQ antibacterial activity against strains of *P. vulgaris* and *S. epidermidis*, we extended the explorations to unveil the proteomic profile of the PQQ-treated bacterial strains by conducting a shutdown proteomic analysis. In this regard, the bacterial strains were treated with PQQ (156.25 μg/mL, half-MIC value) and, subsequently, the cellular proteins from both the PQQ-treated and control bacterial samples were extracted, tryptic digested, and finally subjected to Nano-LC MS/MS analysis. As shown in Figure S4, the analysis of the unsupervised principal components showed significant segregation of the treated group compared to its counterpart control samples, suggesting a change in proteome profile upon PQQ treatment in both bacterial strains. The evaluation of proteomic data for *Proteus vulgaris* revealed 208 unique proteins in the PQQ-treated group and 187 unique proteins in the control group, while a set of 111 shared proteins was presented in both groups (Figure 7A–D). Similarly, the PQQ-treated *S. epidermidis* group exhibited 109 unique proteins, while the control *S. epidermidis* group showed 99 unique proteins, together with an additional 80 proteins which were shared by the PQQ-treated group. Although the shared proteins detected in the two strains displayed a PQQ-dependent abundance (Figure S5), the fold change was not statistically significant between the examined samples. Accordingly, our efforts were directed toward the unique proteins detected in the PQQ-treated groups. To further illustrate the functional meaning of the unique proteins in the PQQ-treated groups, gene ontology (GO) was performed against two databases (Figure 7, supplementary information). Clustering analysis of unique proteins of PQQ-treated *P. vulgaris* and *S. epidermidis* resulted in 17 functional clusters. As shown in Figure 7, PQQ demonstrated the ability to target similar biological processes in both bacterial strains, including ATP metabolic processes, DNA repair processes, membrane and transmembrane transporter proteins, metal-binding proteins, and the oxidative stress response. In this regard, almost 25% of the clusters identified in PQQ-treated *P. vulgaris* proteins were similar to those of PQQ-treated *S. epidermidis*, including membrane and transmembrane transporter proteins. Membrane proteins were the most prominent protein cluster in PQQ-treated *P. vulgaris* and *S. epidermidis* with 19 and 17 proteins, respectively. DNA repair proteins were the second most pronounced affected protein clusters in PQQ-treated *P. vulgaris*, with 14 proteins detected. However, cluster analysis revealed only five proteins related to DNA repair processes in PQQ-treated *S. epidermidis*. Proteins involved in ATP metabolic processes were also markedly affected in strains of *S. epidermidis* (10 proteins) and *P. vulgaris* (7 proteins). Both PQQ-treated bacteria showed noticeable expression for proteins involved in the oxidative stress response, with nine and six proteins in *P. vulgaris* and *S. epidermidis*, respectively. The remaining clusters were dominated by metal ion-binding proteins, and other metabolic processes in both bacteria. Interestingly, PQQ-treated *S. epidermidis* uniquely exhibited the expression of cluster proteins related to quinolone resistance, nitrogen-compound metabolic process, antibiotic resistance, and ribosome biosynthesis. On the other hand, PQQ-treated *P. vulgaris* exclusively showed protein clusters related to RNA metabolism, phospholipid metabolism, pentose catabolic process, choline catabolic process, amino acid metabolism, and flagellar biosynthesis. Taken together, the proteomic analysis indicates that PQQ targets several important cellular processes in bacteria, including membrane proteins, ATP metabolic processes, DNA repair processes, metal-binding proteins, and stress response. These results are in agreement with the findings of TEM analysis, which revealed that PQQ acts by altering the cell membrane and triggering DNA agglomeration and cellular stress.

### 3.6. In Silico Molecular Docking Analyses

#### 3.6.1. *Talaromyces marneffei*

To investigate the molecular effect of PQQ on *T. marneffei*, the binding domain of the Mp1p receptor ligand (PDB ID: *3L1N*) was selected as the target. Mp1p is a cell wall mannoprotein that serves as a lipid transporter between *T. marneffei* and the host [53]. Furthermore, Mp1p is an abundant antigen of the host antibody-mediated response [54,55]. The docking results showed that Mp1p binds to its natural substrate (palmitic acid), making a transient interaction that depends mainly on hydrophobic interactions only (brown color). The predicted chemical interactions between Mp1p docked and palmitic acid did not show any type of hydrogen bonds (Figure S6A). Other chemical interactions were Alkyl with amino acid residues LEU A: 85, LEU A: 145, VAL A: 82, and ILE A: 149. The docking binding score of this interaction was −4.9 kcal/mol, and the ribbon was demonstrated in (Figure S6B) [56]. H-bonds as donors (pink color) and acceptors (green color) were demonstrated in (Figure S6C), and most of the area affected was neutral. Also, charges, ionizability, and SAS interactions (solvent-accessible surface) are illustrated in Figure S6D–G, respectively. The interacted area was also neutral in charges and ionizability.

**Figure 8:**
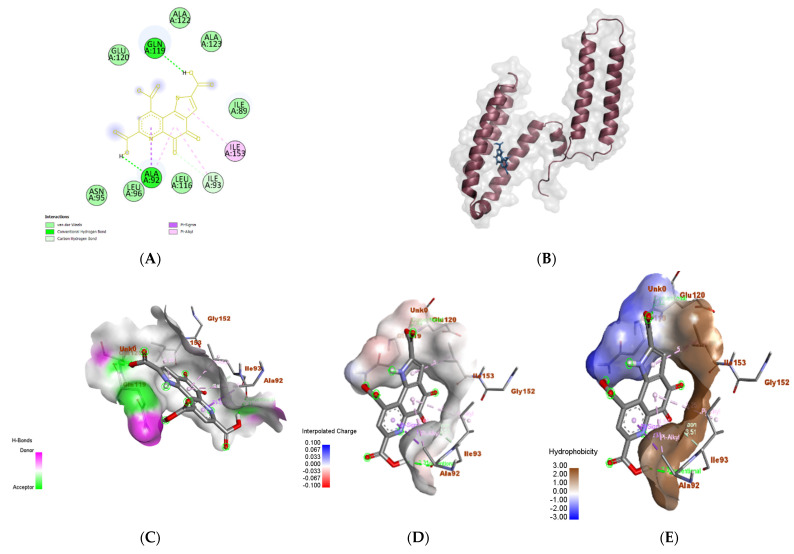

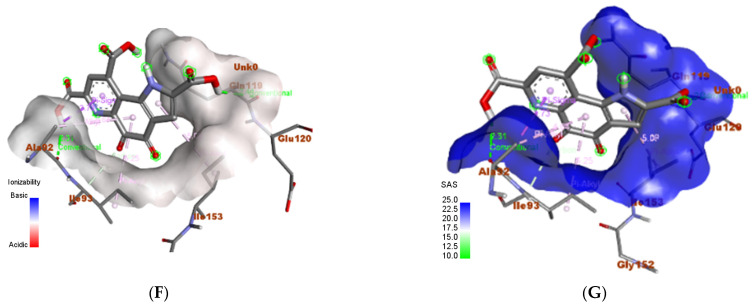
(**A**) 2D chemical interaction of PQQ docked complex with *3L1N* domain, Mp1p receptor, showing 3 hydrogen bonds in green color in the amino acid residues GLN A: 119, ALA A: 92, and ILE A: 93. (**B**) The ribbon demonstrates the PQQ and Mp1p receptor docked complex (binding energy −5.8 kmol/cal) in the active site pocket. (**C**) Surface view showing the hydrogen bond interaction reference ranged from donor in pink and acceptor in green. (**D**) Interpolated charges, reference ranged from the positive charges in blue and the negative charges in red. (**E**) Hydrophobicity, reference ranged from brown color as a hydrophobic and hydrophilicity in blue color. (**F**) Ionizability, reference ranged from basic in blue color, and acidic in red color. (**G**) SAS interactions of the docked complex in blue color; the most interacted area was the solvent-accessible surface.

PQQ established stronger interactions (Figure 8), including two hydrogen bonds with ALA A: 92 and GLN A: 119, in addition to a carbon–hydrogen bond with ILE A: 93 and a pi sigma bond between the aromatic ring and ALA A: 92 (Figure 8A). This interaction exhibited a binding value of −5.8 kcal/mol and showed that the PQQ and Mp1p receptor complex had intensive bounds (Figure 8B). Figure 8C–G showed the donors and acceptors of H-bonds, charges, ionizability, and SAS interactions (solvent-accessible surface), respectively [57]. One edge of the interacted area was negative and hydrophilic. The H bond as a donor was more than the palmitic acid–Mp1p complex. Also, all the areas that interacted were more SAS (surface that is solvent-accessible).

#### 3.6.2. *Proteus vulgaris*

The interaction of PQQ and the cocrystallized ligand; 2-[4-(2-Hydroxyethyl)piperazin-4-ium-1-yl]ethanesulfonate (EPE) with the 5hm C-specific restriction endonuclease PvuRTs1I (PDB ID: *4OQ2*) isolated from *P. vulgaris* was analyzed to investigate the reported antimicrobial effect. The results showed that the enzyme formed multiple conventional hydrogen bonds (ARG A:208, TYR A: 259, THR A: 257) with its 2-[4-(2-hydroxyethyl)piperazin-4-ium-1-yl] ethanesulfonate (EPE), in addition to a carbon–hydrogen bond (GLN A: 182) and a pi–cation bond (HIS A: 166) (Figure S6A) [58]. The interaction complex between the endonuclease binding pocket and EPE resulted in a binding energy of −5.2 kcal/mol (Figure S6B). The area of H bonds was slightly donor-dominant (Figure S6C), charges were neutral (Figure S6D), all areas interacted were hydrophilic (Figure S6E), and basic in their pH (Figure S6F), and half of this area interacted with SAS (Figure S6G).

**Figure 9:**
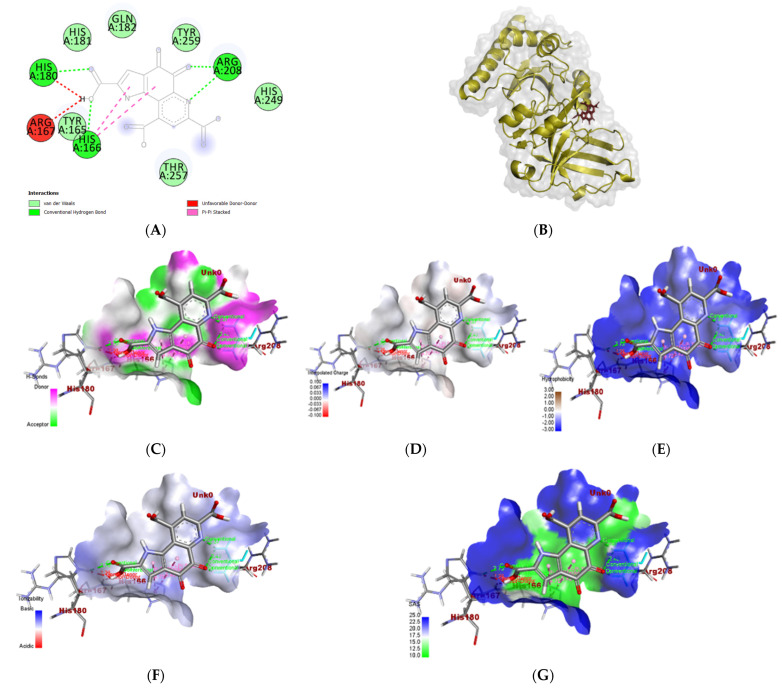
(**A**) 2D chemical interaction of PQQ docked complex with the *4OQ2 domain* showing 4 conventional hydrogen bonds in green in the amino acid residues ARG A: 208, HIS A: 180, and HIS A: 166. (**B**) The ribbon demonstrates the PQQ with the endonuclease-connected complex (binding energy −7 kmol/cal) in the active site pocket. (**C**) Surface view showing the hydrogen bond interaction reference ranged from a donor in pink and acceptor in green. (**D**) Interpolated charges, reference ranged from the positive charges in blue and the negative charges in red. (**E**) Hydrophobicity, reference ranged from brown color for hydrophobic and hydrophilicity in blue color. (**F**) Ionizability, reference ranged from basic in blue color, and acidic in red color. (**G**) SAS interactions of the docked complex reference ranged from high SAS in blue color and low SAS in green color.

All H bonds as donors and acceptors, charges, hydrophobicity, ionizability, and SAS interactions (solvent-accessible surface) (Figure 9) were analyzed to be compared with the PQQ interaction complex. Similarly, PQQ forms two conventional hydrogen bonds with ARG A:208 and another two with HIS A:166 and HIS A:180 (Figure 9A). Additionally, the binding affinity between the same domain and PQQ resulted in a score of −7 kcal/mol, which was less than the previous interaction (Figure 9B). Hydrophobicity, as well as the docked endonuclease–EPE complex, were hydrophilic (Figure S6). However, Figure 9C–G show the H-bond as donors and acceptors, charges, ionizability, and SAS interactions (solvent-accessible surface), respectively [59]. The two docked complexes of endonuclease–EPE and endonuclease–PQQ showed similarity in most of those interactions, but the charges in the endonuclease–PQQ complex were slightly negative compared to in the endonuclease–EPE complex.

#### 3.6.3. *Staphylococcus epidermidis*

For *S. epidermidis*, TcaR was used as a target (PDB ID: 3KP4). TcaR is a negative transcription regulator, and its presence prevents the formation of essential molecules for biofilm formation [60]. In addition to PQQ, the two chains of TcaR were loaded with methicillin, an antibiotic. Methicillin interacted with chain A using two conventional hydrogen bonds (ASN A: 20, HIS B: 42), one carbon–hydrogen bond (ASN A: 45), and a pi–cation bond (Arg a: 110) (Figure S7A). The binding energy for this interaction was −7.5 kmol/cal (Figure S7B). The docked TcaR (chain A) and methicillin complex had hydrophilic hydrophobicity (Figure S7E). On the other hand, Figure S7C–G demonstrated the H-bond as donors and acceptors, charges, ionizability, and SAS interactions, respectively [61]. The H-bonds were more donor than acceptor, charges were slightly negative, and half of the interacted area was SAS interacted.

On the other hand, the docked chain B of TcaR and the methicillin complex depended mainly on hydrophobic interactions (Figure S8). Compared to chain A complex, chain B did not interact with any type of hydrogen bond (Figure S8A). The attached complex of chain B of TcaR and the methicillin complex owned −7.6 kcal/mol of binding energy (Figure S8B). Figure S8C–G, on the other hand, depicted the H bond as donors and acceptors, charges, ionizability, and SAS interactions (solvent-accessible surface), respectively, and were similar to the methicillin–chain A complex [61].

**Figure 10:**
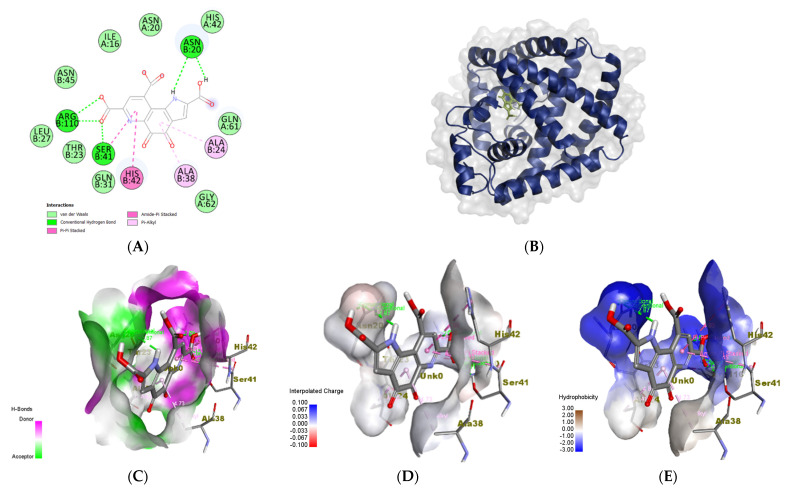

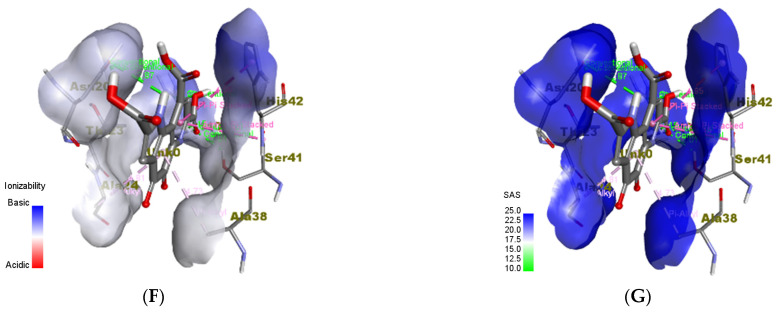
(**A**) 2D diagram showing the chemical interactions between PQQ and 3KP4 domain showing five conventional hydrogen bonds in green color in the amino acid residues ASN B: 20, ARG B: 110, and SER B: 41. (**B**) The ribbon demonstrates the complex docked with 3KP4 domain and PQQ (binding energy −8.8 kmol/cal) in the active site pocket. (**C**) Surface view showing the hydrogen bond interaction reference ranged from donor in pink and acceptor in green. (**D**) Interpolated charges, reference ranged from the positive charges in blue and the negative charges in red. (**E**) Hydrophobicity, reference ranged from brown color for hydrophobic and hydrophilicity in blue color. (**F**) Ionizability, reference ranged from basic in blue color, and acidic in red color. (**G**) SAS interactions of the docked complex in blue color; the most interacted area was the solvent-accessible surface.

For the PQQ docked complex, five conventional hydrogen bonds were formed with chain B involving three residues; ASN B:20, SER B: 41, and ARG B: 110 (Figure 10A). The hydrophobicity of this complex was different from that of the chain A and chain B complexes (Figure 10E). Furthermore, this interaction binding energy was lower than the previous two complexes (−8.8 kcal/mol) (Figure 10B). Additionally, all the H bonds as donors and acceptors, charges, ionizability, and SAS interactions (solvent-accessible surface) were distinct from chain A and chain B complexes (Figure 10C–G) [62]. In particular, the ionizability of the TcaR-PQQ complex was more basic (Figure 10F) and all of the area interacted with SAS (Figure 10G).

All of the previously docked complexes had differences in their docking score, number of H-bonds, hydrophobicity, and distances. The binding affinities of the docked structures showed that PQQ formed more stable complexes with the targeted receptors than those formed with the cocrystallized ligands (Table 1). The high affinity toward these receptors, together with the molecular interactions, emphasizes the antimicrobial effect observed. Additionally, the number of H-bonds and hydrophobicity residues was different among all of the docked complexes. The PQQ in all docked interactions was noted to be the highest in the number of H-bonds, hydrophobicity interaction residues (Table 2), and the lowest binding energy (Table 1). 

## 4. Discussion

PQQ is a water-soluble and heat-stable tricyclic ortho-quinone compound [63]. It has been reported that PQQ has therapeutic capabilities for a variety of diseases, including diabetes mellitus, heart disease, neurological disease, anti-inflammation, antisenescence, and antioxidant effects [64,65,66,67,68,69,70,71]. However, to the best of our knowledge, its antimicrobial capabilities toward specific microorganisms (fungal or bacterial strains) have not been extensively studied and the underlying mechanisms of its antimicrobial activity are still elusive.

Consequently, in this study, the antimicrobial activity of PQQ was evaluated against a variety of known microbes that are pathogenic to either plants or humans. As a result, highly notable antimicrobial activity of PQQ was found against multiple bacterial and fungal strains, including the fungi *T. marneffei* and *Salmonella typhimurium*. On the other hand, the most significant bacterial strains against which PQQ exhibited high activity were *S. epidermidis* among Gram-positive strains, and *P. vulgaris* among Gram-negative strains. *T. marneffei* is a major lung pathogen that is particularly frequent in HIV/AIDS patients [72]. *In vitro* studies show that azole drugs, namely, posaconazole, itraconazole, and voriconazole, possess high antifungal activity against *T. marneffei* [73,74]. However, the high toxicity of the azole drug restricts their usage [75]. Therefore, it is better to use natural extracts, either alone or in combination with other drugs, in therapeutics to reduce the side effects of synthetic drugs. In this regard, a recent study demonstrated the synergistic effect of the combination of berberine, a plant alkaloid with a known antimicrobial effect, with antifungal drugs and reported that berberine improved the *in vitro* effectiveness of antifungal drugs against *T. marneffei* [76]. Similarly, the possible antifungal effect of PQQ presented in this study implies that it could be incorporated in conjunction with other medications to treat *T. marneffei* infections. These results were confirmed by an in-silico docking study, which revealed that PQQ possesses a binding affinity and interactions better than those of cocrystallized palmitic acid. Three H-bonds enhanced the docked Mp1p–PQQ complex, while Mp1p–palmitic acid did not possess any type of H-bonds. The H-bonds as donor and acceptor, therefore, were higher in the PQQ interactions, and the charges varied from neutral in the palmitic acid interactions to slightly negative among the PQQ interactions. Also, the hydrophobicity surface changed to a slightly hydrophilic. Otherwise, the tMp1p–palmitic acid complex was hydrophobic, while the ionizability was the same in the PQQ and palmitic acid complexes. 

Pathogenic strains of *S. epidermidis* have been linked to a variety of skin diseases, such as pediatric atopic eczema, a chronic condition that causes the skin to be dry and itchy, and rosacea, a long-term inflammatory skin condition characterized by flare-ups and remissions [77,78,79]. Lately, three multidrug-resistant strains of *S. epidermidis* have evolved from hospitals and spread globally [80]. These strains have been reported to be resistant to rifampicin, in addition to their reduced susceptibility to vancomycin and teicoplanin. A more recent study isolated *S. epidermidis* strains resistant to methicillin and others with high levels of resistance to other β-lactam antibiotics, namely, penicillin and cefoxitin [81]. Therefore, there is an urgent need for new molecular drugs capable of fighting resistant strains of *S. epidermidis.* PQQ exhibited the ability to induce cellular damage and inhibit *S. epidermidis* replication, indicating its promising capabilities in controlling the growth of *S. epidermidis* at considerable concentrations. Also, *in silico* docking assessments revealed that PQQ interactions were efficiently stabilized by five conventional hydrogen bonds with the lowest binding energy, which indicates that the PQQ interaction is highly stable.

Members of the *Proteus* genus, including *P. vulgaris*, are known to cause healthcare-acquired infections (HAIs) and be responsible for urinary tract infections due to the secretion of the urea-metabolizing enzyme urease [82,83,84]. Furthermore, *P. vulgaris* was identified as a gut pathogen in patients with liver and Crohn’s disease [85,86]. The strains of *P. vulgaris* have been reported to be resistant to β-lactam antibiotics such as ampicillin, tigecycline, chloramphenicol, and cefotaxime, and quinolones such as ciprofloxacin [87,88,89]. Due to its unique chemical scaffolds, PQQ inhibited *P. vulgaris* replication, demonstrating its significant antibacterial potential. Further, a molecular modeling study revealed four conventional hydrogen bonds with a low binding energy. Furthermore, the charges of the interaction appeared to be slightly positive, rather than the neutral charges, in the natural interaction with the PEE.

*Salmonella typhimurium* is a Gram-negative bacterium that causes gastroenteritis in humans and is responsible for the majority of cases of food poisoning in various countries [90]. To progress *S. typhimurium* infection, the bacterium triggers an inflammatory response from the host against the resident microbiota to facilitate colonization of *S. typhimurium* [91]. Multidrug-resistant strains of *S. typhimurium* against ampicillin, streptomycin, sulfonamides, and tetracycline were isolated over the years, indicating the rapid spread of antibiotic resistance in *S. typhimurium* strains [29,92,93,94]. The antimicrobial effect of PQQ reported in the study indicates the potential to use PQQ as a new candidate in the treatment of *S. typhimurium* infections.

**Figure 11:**
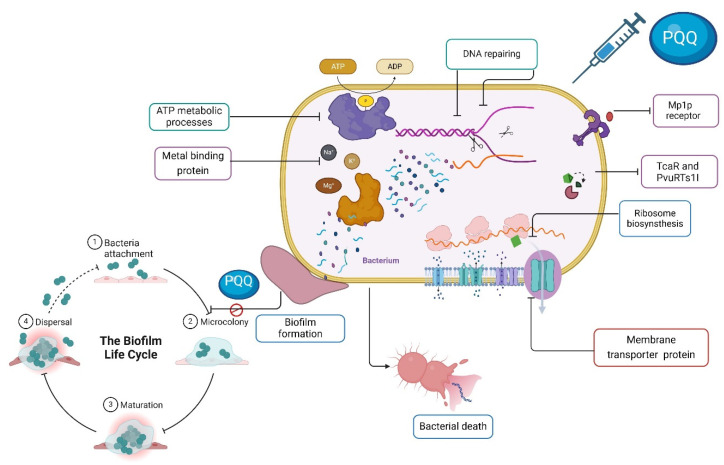
Illustration of the proposed mode of action for the antibacterial activity of PQQ. The diagram depicts the ability of PQQ to (i) disrupt bacterial membranes, ATP metabolic process, biofilm formation, and DNA repair; (ii) to influence the metal ion-binding proteins, impeding the microbial energy production process and the oxidative stress response; and (iii) to efficiently bind to the active site of various microbial targets (Endonuclease PvuRTs1I, TcaR, Mp1p receptor).

Bacterial biofilms consist of multiple microorganisms that are typically enclosed within a polymeric matrix. The presence of biofilms enhances bacterial resistance to antimicrobial treatments and enables their survival under adverse conditions, including evasion of the immune system. Consequently, there has been significant interest in developing more effective drugs to target biofilms. Several promising strategies for biofilm dispersion beyond conventional antibiotics have been explored [95,96]. To contribute to this objective, we conducted further research to investigate the antibiofilm potential of PQQ toward *S. epidermidis*, MRSA, *Micrococcus* sp., *E. cloacae*, *P. vulgaris*, *K. pneumoniae*, and *S. typhimurium*. Our findings demonstrated significant inhibitory activity of PQQ against the tested bacteria in a dose-dependent manner. In the case of Gram-positive bacteria, PQQ demonstrated high inhibitory activity against *MRSA* and *S. epidermidis*, achieving remarkable inhibition rates. For Gram-negative bacteria, PQQ exhibited notable biofilm inhibitory activity against *P. vulgaris* and *S. typhimunium*, resulting in significant reductions in biofilm formation. In line with our findings, a previous study has suggested interference with quorum sensing which is a communication mechanism used by bacteria to coordinate biofilm formation and virulence. PQQ has been suggested to interfere with quorum sensing systems, disrupt bacterial communication, and inhibit the formation of biofilm [97]. Moreover, the chemical structure of PQQ enables it to serve as a cofactor for certain enzymes, particularly those involved in redox reactions. In the context of antibiofilm activity, this redox activity can lead to the generation of reactive oxygen species (ROS). ROS can exert antimicrobial effects by damaging microbial cells and disrupting biofilms. The redox properties of PQQ may contribute to ROS generation, which can enhance the antibiofilm activity [98]. In summary, these findings highlight the strong antimicrobial activity of PQQ, particularly in targeting the formation of biofilms by the tested bacterial species (Figure 11).

The application of proteomic analysis tools demonstrated the ability to identify, validate, and explore the expression of the proteins involved in triggering bacterial death [99,100,101]. The assessment of the proteomic data for *Proteus vulgaris* treated with PQQ and *S. epidermidis* showed 208 and 109 unique proteins. The findings of the gene ontology (GO) enrichment analysis of PQQ-treated bacteria revealed that PQQ influences essential biological processes within the proteomes of both *P. vulgaris* and *S. epidermidis* and provides a possible mode of action for the antibacterial activity of PQQ (Figure 11). Interestingly, most of the proteins identified in treated bacteria were related to membrane and transmembrane transporter proteins, highlighting the disruption of bacterial membranes. These findings further align with the potential antibiofilm activity observed for PQQ toward the examined bacterial strains. Overexpression of membrane proteins may mediate various nonphysiologically bacterial responses, such as membrane disruption [99]. The proteomic profile also revealed the expression of DNA repair proteins in response to PQQ treatment (Figure 11). These results further support the findings observed by TEM analysis. The expression of such proteins is a key event in cell defense mechanisms to cytotoxic agents [102,103]. Several pieces of evidence have been reported on the DNA damage effect of quinones through interaction with DNA topoisomerase, which induces the SOS response of bacteria [104,105]. This global response to DNA damage includes the induction of the DNA repair pathway through the expression of many proteins that promote DNA integrity in bacteria [106,107]. Consistently, induction of the SOS response was observed with quinolone treatment [108,109]. The DNA repair and stress response pathways use ATP as an energy source. These facts may interpret the activation of ATP metabolic proteins in *P. vulgaris* and *S. epidermidis* treated with PQQ [110]. It has been widely reported that the antibacterial mechanism of action of quinolines depends mainly on the inhibition of ATP synthesis activity by targeting the c-Ring of ATP synthase [111,112,113]. Targeting energy metabolism has been the mainstay of a new promising approach in antibacterial drug discovery [113,114]. A variety of quinolines exert their antimicrobial and anticancer activity by inducing oxidative stress by disrupting the redox potential of the cell [115,116]. Likewise, treatment of *P. vulgaris* and *S. epidermidis* with PQQ also activates the expression of oxidative stress response proteins, as revealed in the proteomic analysis in the present study (Figure 11). The expression of these groups of proteins was associated with the activation of metal ion-binding proteins. PQQ, as a tricyclic orthoquinone, acts as a redox cofactor for several bacterial reactions involved in energy production, metabolism, and other biological processes [117]. Such activation may suggest that PQQ binds to metals at the active site of oxidoreductases, which interferes with their catalytic functions and leads to oxidative stress and consequent cell death [116]. PQQ-treated *P. vulgaris* unquietly displayed the expression of phosphorylation protein. This group of proteins plays a pivotal role in regulating several mechanisms in bacterial responses against antibacterial agents [118,119]. Remarkably, proteomic analysis of PQQ-treated *S. epidermidis* provides evidence that PQQ induces the expression of proteins associated with resistance to quinolines and antibiotic resistance. Recent studies showed that the resistance rate to quinolines of *S. epidermidis* was increased in normal conjunctival microbes [120,121].

Based on the presented findings, we suggest that the antimicrobial activity of PQQ can be attributed to multiple mechanisms. Firstly, PQQ inhibits the activity of essential microbial enzymes for survival and growth, such as bacterial alcohol dehydrogenase, thus disrupting crucial metabolic pathways and inhibiting microbial growth [122,123]. Secondly, PQQ disrupts the electron transport chains in microorganisms, leading to the disruption of energy production and a reduction in microbial viability. Furthermore, PQQ interferes with quorum sensing systems, disrupting the communication between bacterial cells, and reducing the expression of virulence factors and biofilm formation [124]. Furthermore, PQQ exhibits immunomodulatory effects, enhancing the activity of the immune system and aiding in the elimination of microbial pathogens [125]. Finally, PQQ shows the ability to target several biological processes in bacteria, including ATP metabolic processes, the bacterial membrane, DNA repair processes, metal-binding proteins, and stress response (Figure 11). It is important to note that the exact mode of action may vary depending on the targeted microorganism, and further research is needed to fully comprehend the precise mechanisms underlying PQQ’s antimicrobial activity.

## 5. Conclusions

In conclusion, the meticulous investigation of PQQ’s antimicrobial activity against a diverse panel of 29 pathogenic microbes, encompassing both fungal and bacterial strains, unveils its profound and distinctive antifungal properties. Specifically, PQQ shows remarkable effectiveness against notorious pathogens such as *S. racemosum*, *T. marneffei*, *C. lipolytica*, and *T. rubrum*. The varying MIC values across fungal strains highlight the importance of considering pathogen-specific sensitivities, an essential aspect in the rational design of future antimicrobial interventions. Furthermore, PQQ exhibits a conspicuous antibacterial activity that effectively hinders the growth of Gram-positive bacteria, most notably *S. epidermidis* and *MRSA* strains. Although certain bacterial strains, such as *S. aureus*, exhibit somewhat elevated MIC values, the overall broad-spectrum antimicrobial potential of PQQ remains undeniably noteworthy. The discovery of PQQ’s ability to mitigate biofilm formation represents a crucial advancement in addressing recalcitrant and tenacious microbial infections often associated with biofilms. Significant inhibitory effects against biofilms formed by MRSA, *S. epidermidis*, and *P. vulgaris* accentuate the capacity of PQQ to combat challenging biofilm-related infections, further increasing its value as a therapeutic option. Through the lens of TEM, PQQ’s mode of action is illuminated, unveiling the captivating spectacle of structural disruption and metabolic impairment within bacterial cells. The prowess of PQQ in compromising cell membrane integrity and engendering leakage of cytoplasmic contents reinforces the evidence for its potent antimicrobial efficacy. These findings were further demonstrated by proteomic analysis, showing that PQQ can target various biological processes in bacteria, including membrane proteins, ATP metabolic processes, DNA repair processes, metal-binding proteins, and stress response. Moreover, in-depth molecular modeling investigations unravel the intricate interactions between PQQ and key microbial targets, including mannoprotein Mp1P, transcriptional regulator TcaR, and endonuclease PvuRTs1I, providing a profound molecular rationale for its antimicrobial actions. Collectively, these results strongly support the potential of PQQ as a promising candidate for incorporation into future antimicrobial pharmaceutical formulations. Its potent antifungal and antibacterial activities, combined with its ability to inhibit biofilm formation and target several critical biological processes, establish PQQ as an effective and versatile agent with broad-spectrum antimicrobial activity.

## Figures and Tables

**Table 1:** Binding affinities of PQQ toward a set of targeted proteins.

Receptor (Organism)	Ligand	Binding Affinity (kcal/mol)
Mp1p receptor (*T. marneffei*)	Palmitic Acid	−4.9
	PQQ	−5.8
Endonuclease PvuRTs1I (*P. vulgaris*)	EPE	−5.2
	PQQ	−7
TcaR (*S. epidermidis*)	Methicillin (A)	−7.5
	Methicillin (B)	−7.6
	PQQ	−8.8

**Table 2:** The list of hydrophobic and H-bond interactions of PQQ and cocrystallized ligands toward the target proteins.

**Protein**	**Ligand**	**Hydrophobic Residues**	Distance	H-Bond Residues	Distance
Mp1p receptor (*T. marneffei*)	Palmitic Acid	VAL82 LEU85 LEU145 ILE149	4.6958 4.58823 4.64399 5.25498	-	-
	PQQ	ALA92:CB ALA92 ILE93 ILE153	3.72868 4.64191 5.24657 5.082	ALA92 GLN119 ILE93	2.30691 2.44378 3.51331
Endonuclease PvuRTs1I (*P. vulgaris*)	EPE	-	-	ARG208 THR257 TYR259 HIS181	2.19337 2.92358 2.20303 3.77114
	PQQ	A:HIS166 A:HIS166	5.42052 4.70423	HIS166 HIS180 ARG208 ARG208 ARG208	2.30924 2.75025 1.89771 2.36602 2.49164
TcaR (*S. epidermidis*)	Methicillin (A)	HIS42:HD2 HIS42 ALA24 ALA38 VAL63 HIS42 HIS42	2.89919 5.13187 4.14757 4.03449 5.03687 4.38379 4.62078	ASN20 HIS42 ASN20 ASN45 ARG110	2.92935 2.27991 2.76373 3.05598 4.19579
	Methicillin (B)	HIS42 VAL63 HIS42	3.94929 4.54622 5.12969	-	-
	PQQ	HIS42 SER41:C,O;HIS42:N ALA24 ALA38	4.95108 5.44787 4.81024 4.72874	SER41 ARG110 ARG110 ASN20 ASN20	2.01432 2.15174 2.28594 2.87169 2.01751

## Data Availability

This study’s original contributions are included in this article.

## Supplementary Materials

The following supporting information can be downloaded at: https://www.mdpi.com/article/10.3390/biom14081018/s1, Figure S1: Growth inhibition zones of *Penicillium marneffeii* and *Trichophyton rubrum* after treatment with Ketoconazole; Figure S2: Growth inhibition zones of *Staphylococcus epidermidis*, *Methicillin-Resistant Staphylococcus aureus* (MRSA), and *Staphylococcus aureus* after treatment with Gentamycin; Figure S3: Growth inhibition zones of *Proteus vulgaris*, *Salmonella typhimurium*, and *Enterobacter cloacae* after treatment with Gentamycin; Figure S4: Principal component analysis (PCA) between treated and control groups for *Proteus vulgaris* and *Staphylococcus epidermidis*; Figure S5: Scatter plot showing the correlation of the protein abundances between the treated and control groups in *Proteus vulgaris* and *Staphylococcus epidermidis*; Figure S6: Interaction of palmitic acid with the ligand binding domain of the Mp1p receptor; Figure S7: Interaction of the endonuclease with EPE binding pocket; Figure S8: Interactions between TcaR (chain A) and methicillin; Figure S9: Interactions between chain B of TcaR and methicillin.; Table S1: The antifungal activity of the ketoconazole as minimum inhibitory concentration (MIC) in μg/mL toward the examined fungal strains using the diffusion agar technique; Table S2: The antibacterial activity of the gentamycin as minimum inhibitory concentration (MIC) in μg/mL toward the examined bacterial strains using the diffusion agar technique.
