# Supplementary material for: Novel Insights into the Antimicrobial and Antibiofilm Activity of Pyrroloquinoline Quinone (PQQ); In Vitro, In Silico, and Shotgun Proteomic Studies
Source: Biomolecules. 2024 Aug 16;14(8):1018. doi: 10.3390/biom14081018 (PMC11352295; doi:10.3390/biom14081018)
Supplement: Supplementary file 1 [file biomolecules-14-01018-s001.zip › biomolecules-3056175-supplementary.pdf]

<u>Supplementary Materials</u>

Novel Insights into the Antibacterial, Antifungal, and Antibiofilm Activity of Pyrroloquinoline quinone (PQQ); *in vitro*, *in silico* and Shotgun Proteomic Studies

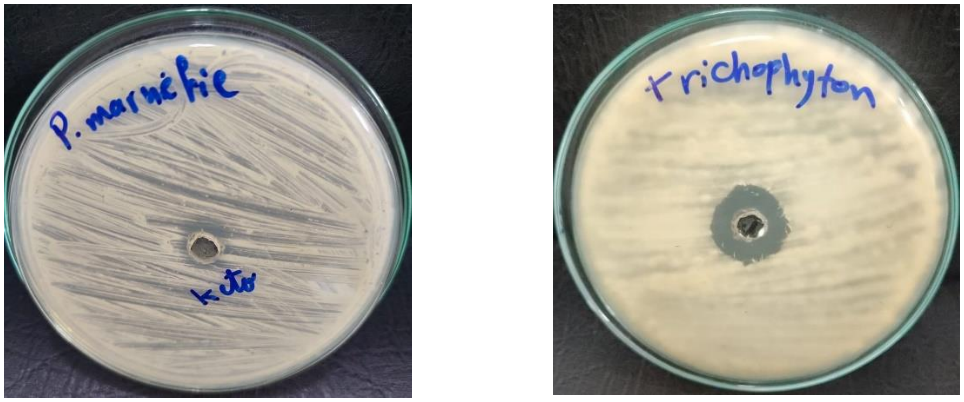

(A) **(B)**

Figure S1. (A-B) Growth inhibition zones of *Penicillium marneffeii* (**A**) and *Trichophyton rubrum* (**B**) after treatment with Ketoconazole.

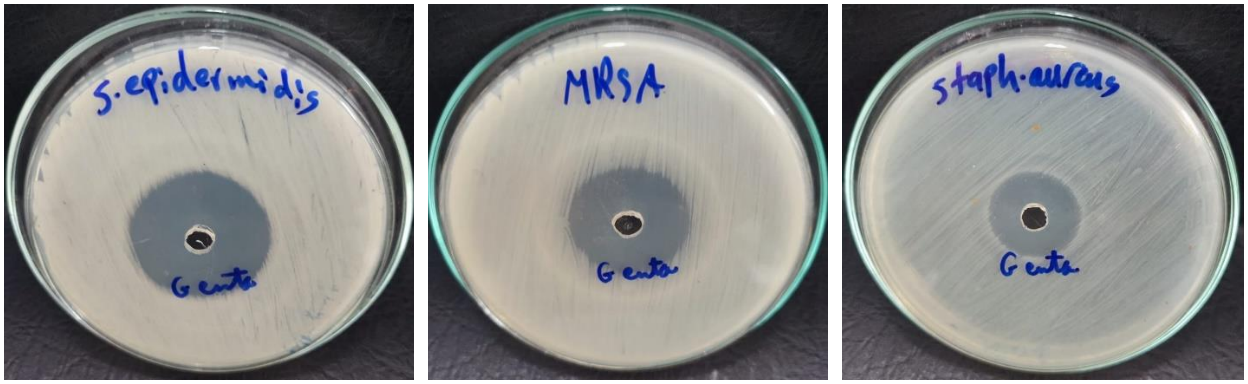

(A) **(B)** **(C)**

Figure S2. (A-C) Growth inhibition zones of *Staphylococcus epidermidis* (**A**), *Methicillin-Resistant Staphylococcus aureus* (MRSA) (**B**), and *Staphylococcus aureus* (**C**) after treatment with Gentamycin.

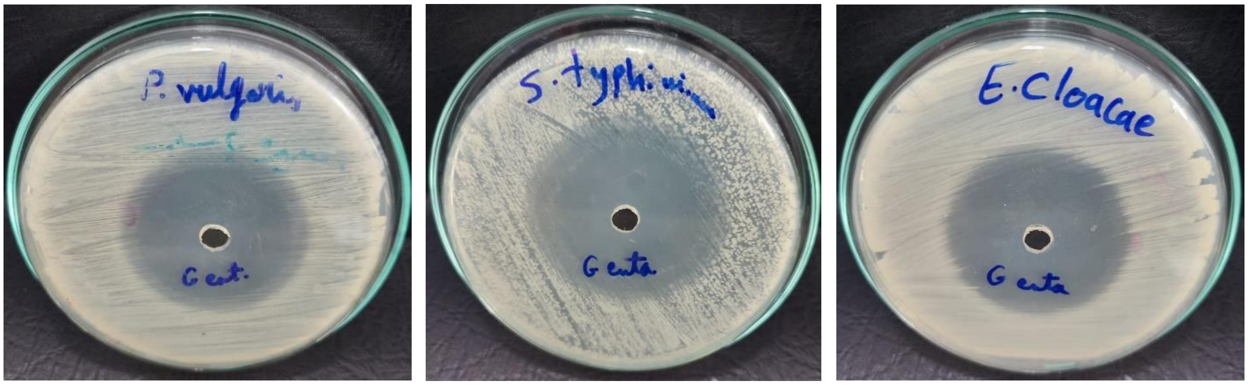

(A) **(B)** **(C)**

Figure S3. (A-C) Growth inhibition zones of *Proteus vulgaris* (**A**), *Salmonella typhimurium* (**B**), and *Enterobacter cloacae* (**C**) after treatment with Gentamycin**.**

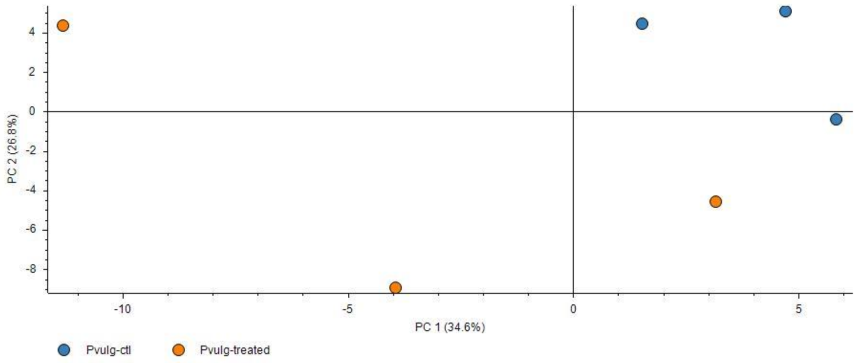

(A)

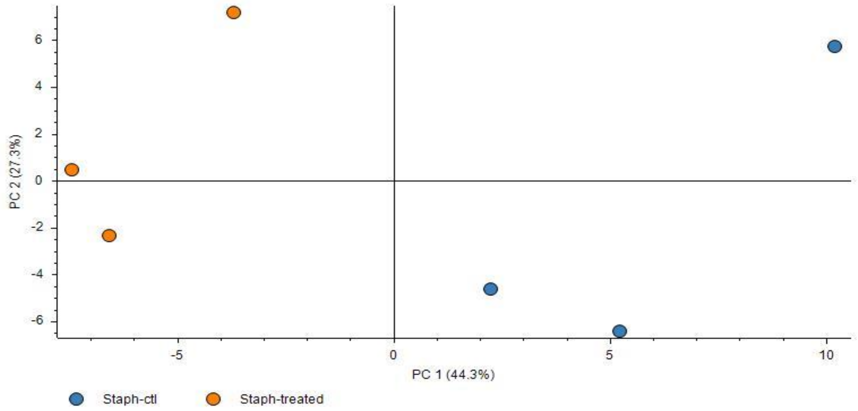

(B)

Figure S4: Principal component analysis (PCA) between treated and control groups for *Proteus vulgaris* **(A)** and *Staphylococcus epidermidis* **(B)**.

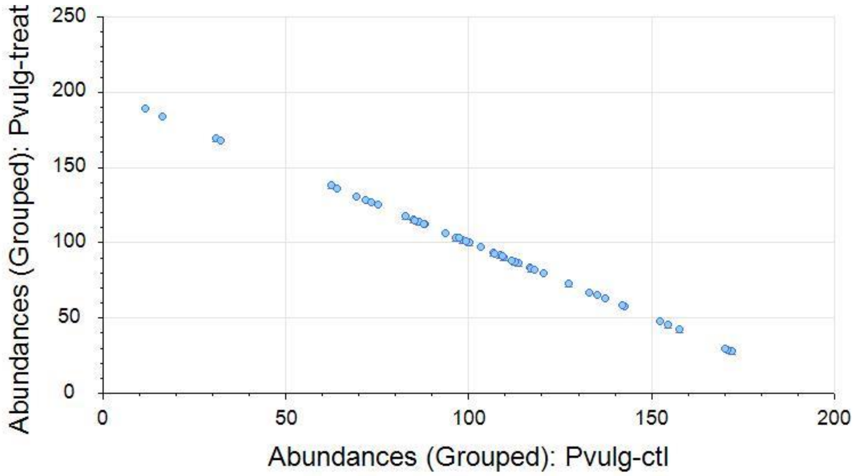

(A)

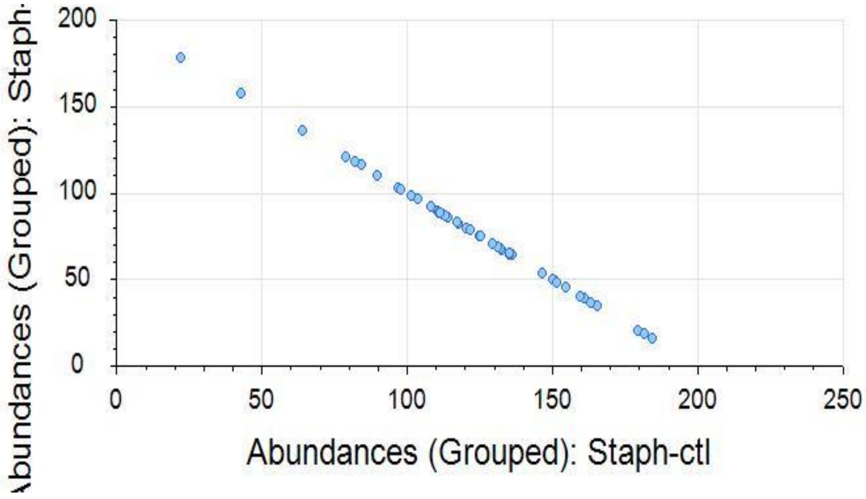

(B)

Figure S5: Scatter plot showing the correlation of the protein abundances between the treated and control groups in *Proteus vulgaris* **(A)** and *Staphylococcus epidermidis* **(B).**

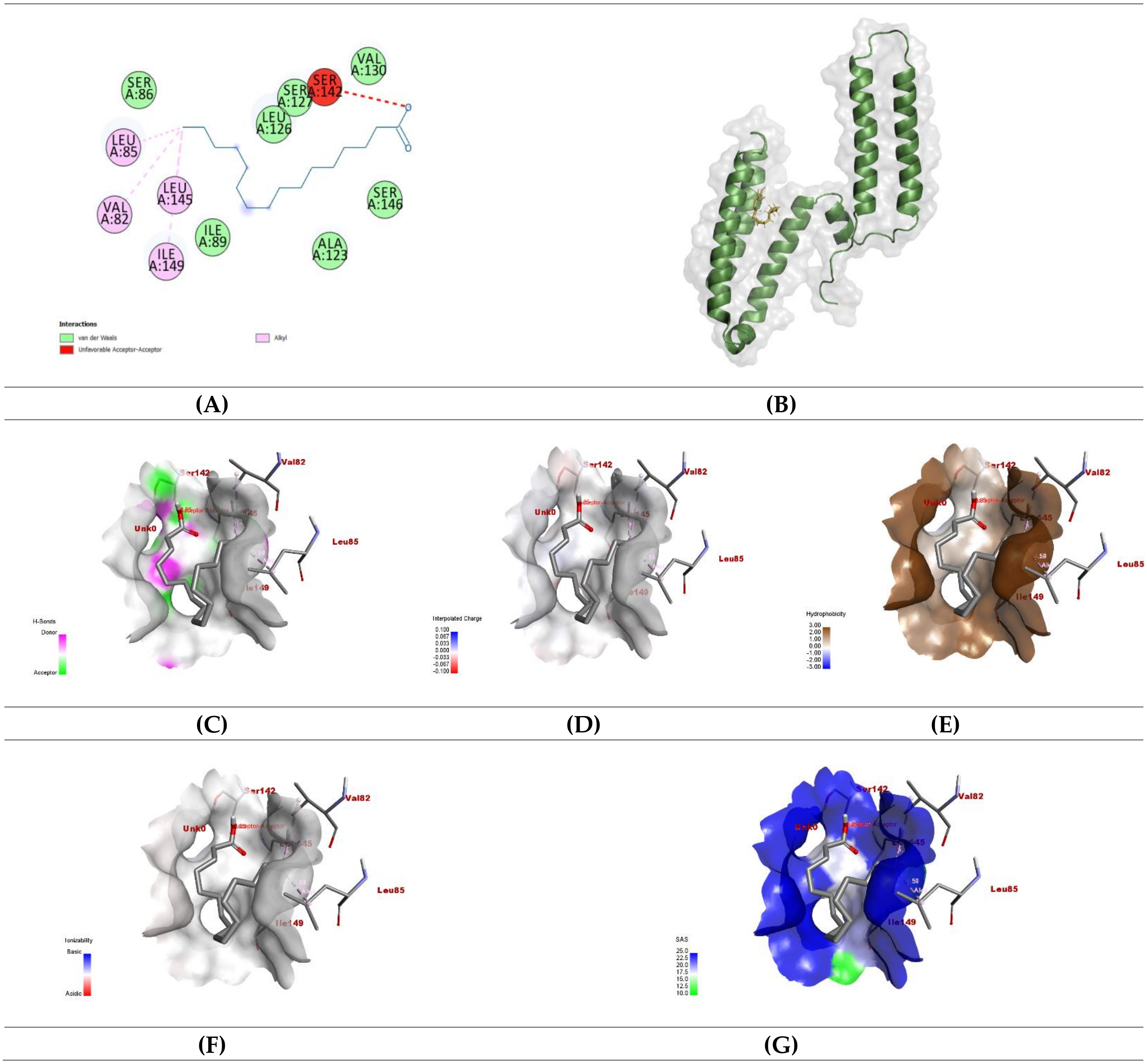

Figure S6. (A) 2D chemical interaction of palmitic acid with the ligand binding domain of the Mp1p receptor. (B) The ribbon demonstrates the palmitic acid and Mp1p receptor docked complex (binding energy -4.9 kmol/cal). (C) Surface view showing the hydrogen bond interaction. (D) Interpolated charges. (E) Hydrophobicity. (F) Ionizability. (G) SAS interactions of the docked complex.

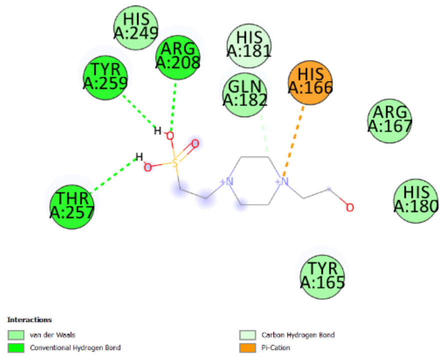

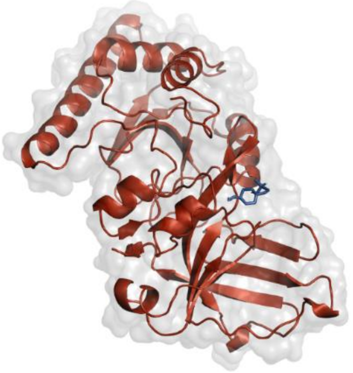

(A) **(B)**

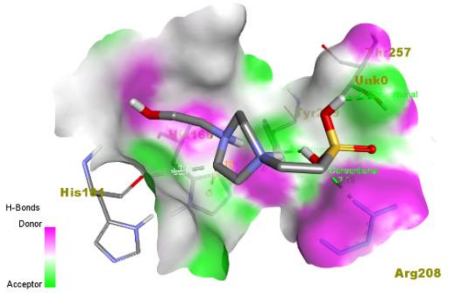

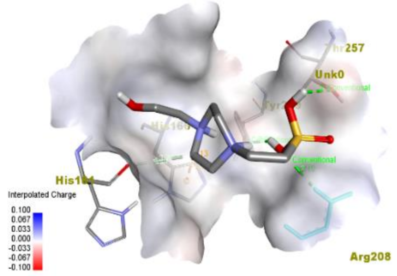

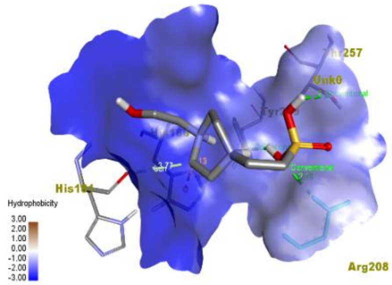

(C) **(D)** **(E)**

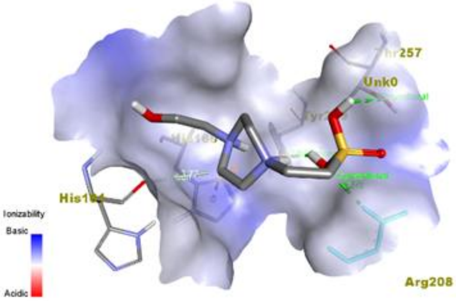

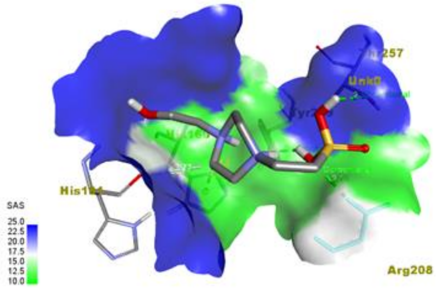

(F) **(G)**

Figure S7. (A) 2D chemical interaction of the endonuclease with EPE. (B) The ribbon demonstrates the endonuclease with EPE docked complex (binding energy -5.2 kmol/cal). (C) Surface view showing the hydrogen bond interaction. (D) Interpolated charges. (E) Hydrophobicity. (F) Ionizability. (G) SAS interactions of the docked complex.

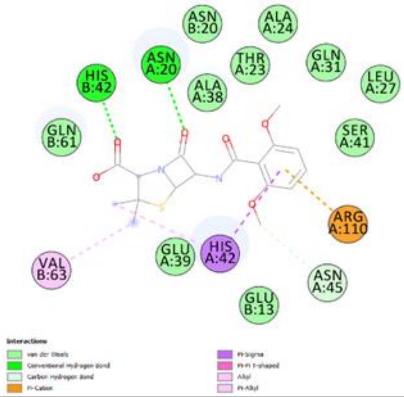

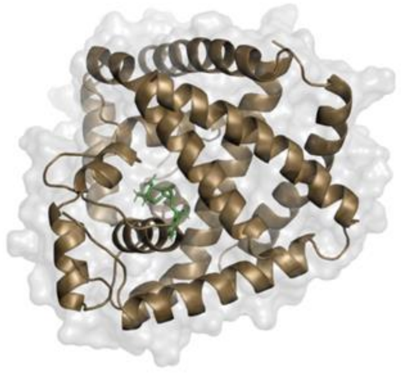

(A) (B)

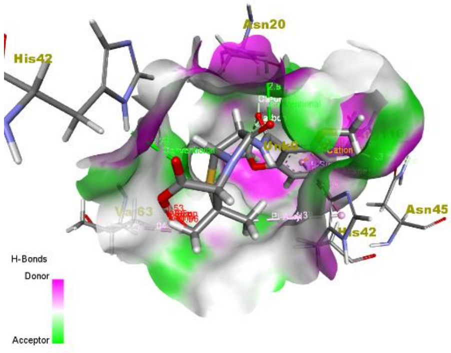

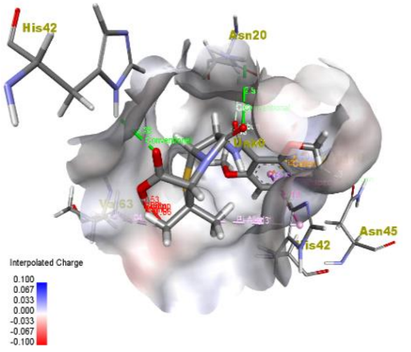

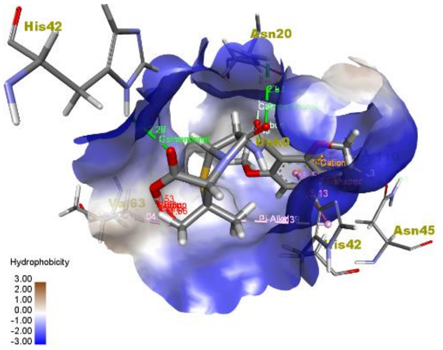

(C) (D) (E)

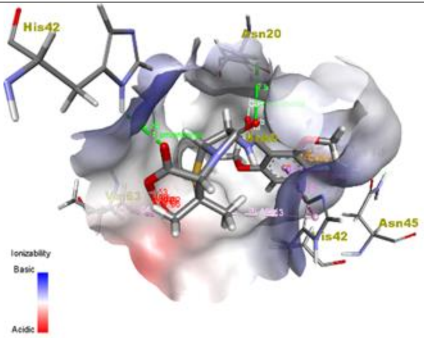

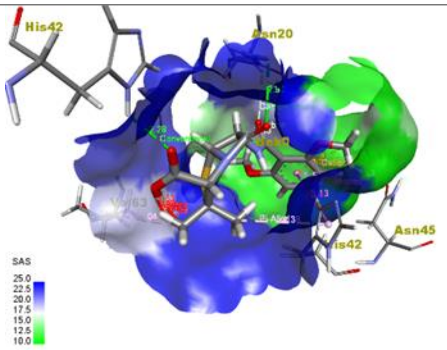

(F) (G)

Figure S8. (A) 2D diagram showed the chemical interactions between TcaR (chain A) and methicillin. (B) The ribbon demonstrates the TcaR (chain A) and methicillin docked complex (binding energy -7.5 kmol/cal). (C) Surface view showed the hydrogen bond interaction. (D) Interpolated charges. (E) Hydrophobicity. (F) Ionizability. (G) SAS interactions of the docked complex.

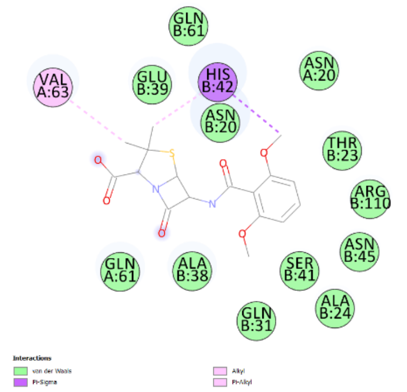

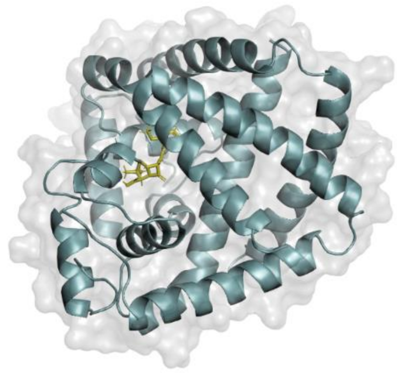

(A)	(B)

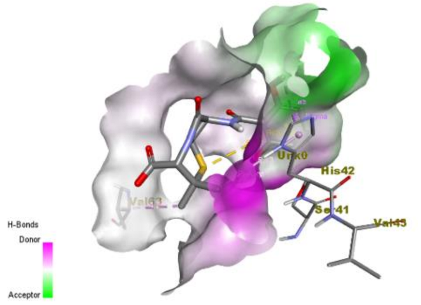

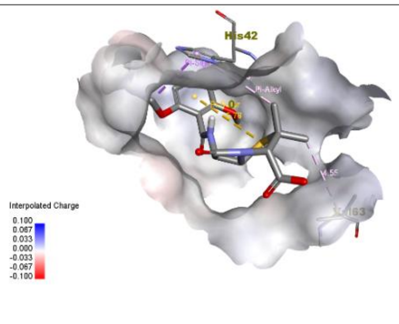

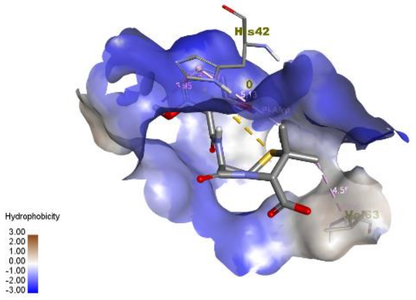

(C)	(D)	(E)

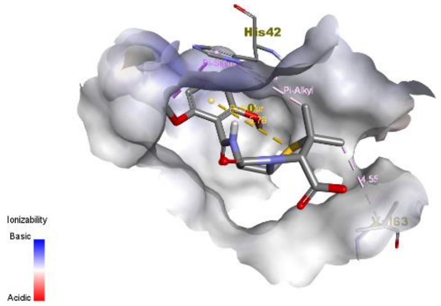

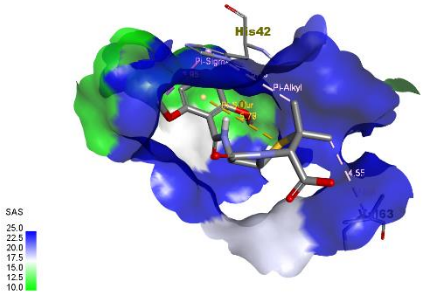

(F)	(G)

Figure S9. (A) 2D diagram showing the chemical interactions between chain B of TcaR and methicillin. (B) The ribbon demonstrates the chain B of TcaR and methicillin docked complex (binding energy -7.6 kmol/cal). (C) Surface view showing the hydrogen bond interaction. (D) Interpolated charges. (E) Hydrophobicity. (F) Ionizability. (G) SAS interactions of the docked complex.

Table S1: The antifungal activity of the ketoconazole as minimum inhibitory concentration (MIC) in µg/mL toward the examined fungal strains using the diffusion agar technique.

Fungal strain	Ketoconazole	
	MIC	MFC
Syncephalastrum racemosum RCMB 016001 (1)	0.05	Cidal
Penicillium marneffeii (RCMB 001022)	0.35	Cidal
Candida lipolytica RCMB 005007(1)	2	Cidal
Trichophyton rubrum (RCMB 025002)	1	Cidal

Table S2: The antibacterial activity of the gentamycin as minimum inhibitory concentration (MIC) in µg/mL toward the examined bacterial strains using the diffusion agar technique.

Bacterial strain	Gentamycin	
	MIC	MBC
Staphylococcus aureus ATCC 25923	1	Cidal
Staphylococcus epidermidis RCMB 009 (2)	0.8	Cidal
Micrococcus sp. RCMB 028 (1)	1.5	Cidal
Methicillin-Resistant Staphylococcus aureus (MRSA) ATCC 4330	4	Cidal
Enterobacter cloacae RCMB 001 (1) ATCC 23355	60	Cidal
Salmonella typhimurium RCMB 006 (1) ATCC 14028	0.5	Cidal
Proteus vulgaris RCMB 004 (1) ATCC 13315	3	Cidal
Serratia marcenscens 007001	2	Cidal
